# Metallic nanoplatforms for COVID-19 diagnostics: versatile applications in the pandemic and post-pandemic era

**DOI:** 10.1186/s12951-023-01981-5

**Published:** 2023-08-04

**Authors:** Yuqing Li, Jingqi Chen, Jinchao Wei, Xueliang Liu, Lu Yu, Linqi Yu, Ding Ding, Yu Yang

**Affiliations:** 1 Institute of Molecular Medicine (IMM), School of Medicine, Renji Hospital, Shanghai Jiao Tong University, Shanghai, 200127 China; 2 Institute of Functional Nano & Soft Materials (FUNSOM), Jiangsu Key Laboratory for Carbon-Based Functional Mate-Rials & Devices, Soochow University, Suzhou, 215123 China; 3 Department of Immunization Program, Jing’an District Center for Disease Control and Prevention, Shanghai, 200072 China; 4 State Key Laboratory of Quality Research in Chinese Medicine, Institute of Chinese Medical Sciences, University of Macau, Macau, China

**Keywords:** COVID-19, Nanomaterials, Diagnosis, Surveillance

## Abstract

The COVID-19 pandemic, which originated in Hubei, China, in December 2019, has had a profound impact on global public health. With the elucidation of the SARS-CoV-2 virus structure, genome type, and routes of infection, a variety of diagnostic methods have been developed for COVID-19 detection and surveillance. Although the pandemic has been declared over, we are still significantly affected by it in our daily lives in the post-pandemic era. Among the various diagnostic methods, nanomaterials, especially metallic nanomaterials, have shown great potential in the field of bioanalysis due to their unique physical and chemical properties. This review highlights the important role of metallic nanosensors in achieving accurate and efficient detection of COVID-19 during the pandemic outbreak and spread. The sensing mechanisms of each diagnostic device capable of analyzing a range of targets, including viral nucleic acids and various proteins, are described. Since SARS-CoV-2 is constantly mutating, strategies for dealing with new variants are also suggested. In addition, we discuss the analytical tools needed to detect SARS-CoV-2 variants in the current post-pandemic era, with a focus on achieving rapid and accurate detection. Finally, we address the challenges and future directions of metallic nanomaterial-based COVID-19 detection, which may inspire researchers to develop advanced biosensors for COVID-19 monitoring and rapid response to other virus-induced pandemics based on our current achievements.

## Introduction

Coronavirus disease (COVID-19) which is caused by the infection of severe acute respiratory syndrome coronavirus 2 (SARS-CoV-2) has been broken out as a pandemic at the end of 2019 [1, 2]. As of December 2022, this pandemic has officially spread to more than 200 countries, infected 640 million people, and resulted in 6.6 million deaths globally [3]. Meanwhile, there is a susception that the total number of reported infected cases is underestimated because of the asymptomatic carriers and data missing of some regions. Thanks to the generation of COVID-19 vaccines [4, 5], most countries have entered the post-pandemic era, in which people can try to face the SARS-CoV-2 in their daily life and learn to live with it. Basically, the highly infective SARS-CoV-2 mainly spreads by the small liquid particles containing the virus via close contact including cough, sneeze, speak, singing, or breathing [6, 7]. Due to the nature of virus, SARS-CoV-2 is constantly changing over time and leads to the emergence of new variants that possess new characteristics [8–10]. For example, the delta variant (B.1.617.2) that was first documented in India in Oct-2020, was demonstrated more contagious and might cause more severe illness than previous variants in unvaccinated people [11, 12]. Nowadays, variant Omicron (B.1.1.529) has been acknowledged to be the currently circulating variant of concern by WHO and accounts for the majority of newly identified COVID-19 cases world widely [13, 14]. These new variants released pressures on the public health system and challenged the whole human society. To fight against the SARS-CoV-2 and its variants in the post pandemic era, COVID-19 tests, in addition to vaccine administrations, plays a critical role to mitigate the pandemic by identifying infected individuals, then further prevent person-to-person transmission [15–18]. To this regard, effective, accurate as well as time-saving testing is greatly helpful to decide whether the quarantine is needed and the controlling of the virus transmitting.

COVID-19 infection often shows similar symptoms with influenza such as fever, cough, shortness of breath, and other symptoms. Thus, simply determining a new case based on the symptoms is not adequate. Chest computed tomography (CT) were preliminarily used as the diagnostic method, since the presence of pulmonary consolidation in CT images indicated the onset of the COVID-19 pneumonia [19]. However, the CT was still inaccurate when determining some asymptomatic carriers and patients with mild symptoms, whose consolidation lesions were hard to identify. Instead, the diagnosis relying on molecular recognition would be more rational and precise.

**Fig. 1:**
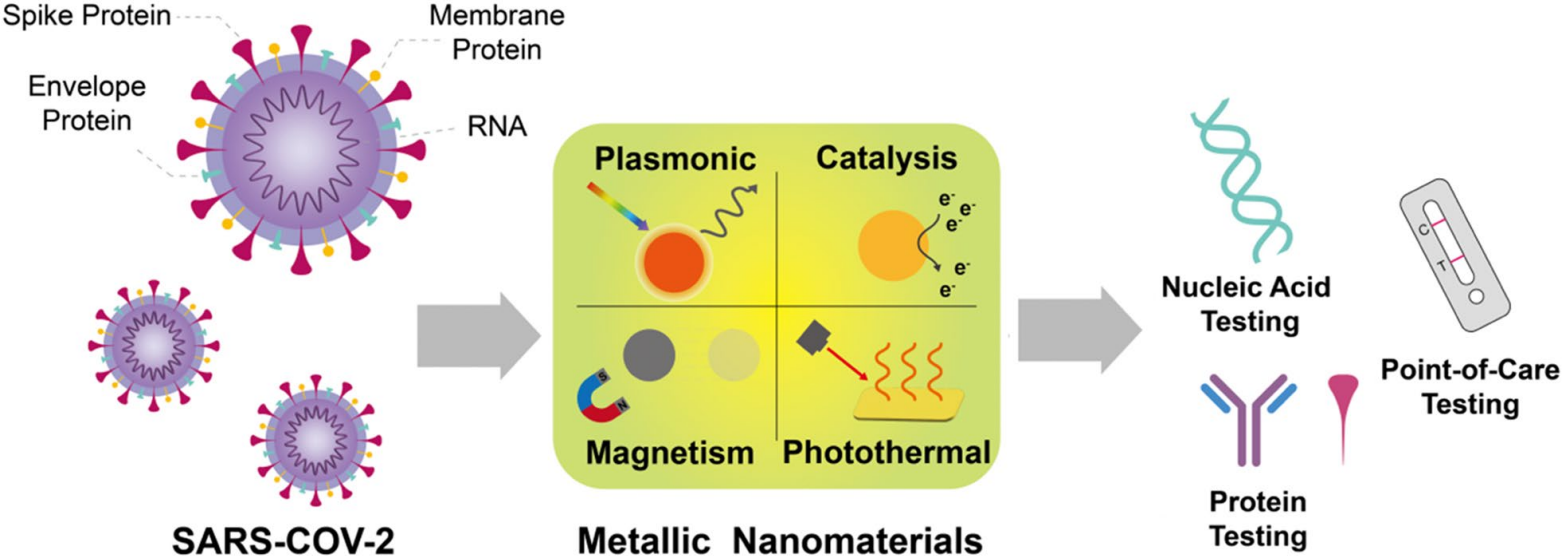
Schematic illustration of the SARS-CoV-2 virus structure and respective diagnostic applications based on the metallic nanomaterials’ properties

Structurally, SARS-CoV-2 is similar to other coronaviruses from the family of *Coronaviridae*, bearing a positive-sense single-stranded RNA (around 30,000 nucleotides) that is in charge of the translation of virus genetic materials into proteins in the infected cells [20]. Besides, SARS-CoV-2 has four structural proteins and named spike protein (S protein), envelope protein (E protein), membrane protein (M protein), and nucleocapsid protein (N protein) (Fig. 1) [21]. Based on the biological functions of these four proteins, the S protein is responsible for allowing entrance of host cells, the smallest E protein helps the dissemination and replication process, the M protein holds the structure of the virion, as well as the shape and size of the virus, while the N protein plays important role in the virus replication. Therefore, the detecting strategies can vary a lot based on different targeting genes and proteins. To realize a fast and sensitive clinical diagnose of COVID-19, it is essential to identify specific biomarkers that are concurrently present during the infection. Basically, the testing of SARS-CoV-2 could be categorized into direct and indirect detections [22]. The direct method refers to the detection of as mentioned RNA or proteins involved in the structure of the virus, while the indirect ways include tracing excretive antibodies and cytokines that are generated during inflammatory and immune processes (Table 1).

**Table 1:** The comparison of different detecting strategies

	Viral tests		Antibody tests
	Nucleic acid amplification tests (NAATs)	Antigen tests	
Analyte detected	Viral RNA	Viral antigens	Antibodies
Sample types	Upper respiratory specimens^a^	Upper respiratory specimens or serum	Serum/plasma or whole blood specimens
Turnaround time	Within 24 h^b^	Within 15–30 min	Within 24 h^d^
Sensitivity	Generally high^c^	Pending on the infection course	Varies by test
Intended use	Detection of current infection		Detection of previous infection
Testing scenarios	Diagnostic and screening testing		Public health surveillance testing
Cost^e^	~ $ 2.3	~ $ 0.85	~ $ 5.8

^a^Aside from nasal and nasopharyngeal, NAATs sample types also including oropharyngeal, sputum and saliva

^b^Few of the POC test-based NAATs could be finished in 15 min

^c^POC test-based NAATs show moderate high sensitivity

^d^Some POC test-based antibody testing could be finished in 15 min

^e^The cost is based on the detection price in China in 2023 with health insurance coverage

Taking advantage of molecular biology techniques, for example, the viral RNA can be detected through a quantitative reverse transcription polymerase chain reaction (RT-PCR) in hours [23]. Although the PCR and most immunoassay-based methods are the standard diagnostic procedures for the SARS-CoV-2 infection, they are quite time-consuming and costly [24]. To realize a faster, easier, sensitive as well as more affordable diagnosis, biosensors attract attentions therefore they are actively explored [25]. Among them, the metal-based nanoplatforms are mostly studied, owing to their high sensitivity in sensing, easy to use, inexpensiveness and easy disposability [26, 27]. The optical, electrochemical, and magnetic properties of nanometals and their bioconjugation abilities enable researchers to fabricate a variety of sensors for detecting the SARS-CoV-2 with improved sensitivity and specificity.

In this review, we highlight recent research progress on using metallic nanomaterials in the diagnosing and surveilling of COVID-19, providing a critical discussion about their usability and performance compared with conventional diagnostic strategies. The most representative metallic nanomaterials such as gold nanoparticles, magnetic nanoparticles, lanthanides nanoparticles and quantum dots are reviewed; and point-of-care testing strategies relying on these nanomaterials are also covered. Then, to demonstrate how to cope with constantly changing variants, we also discussed and offer insights in this part. Finally, current challenges and future directions of metallic nanomaterials-based diagnosis and surveillance of COVID-19 are summarized and suggested as well. We reason that this review can provide insights into the development of metal-based nanoplatforms for SARS-CoV-2 surveillance in both the pre- and post-pandemic era, and in a larger sense, can provide ideas for other researchers to design more viral elements associated biosensors for disease monitoring.

## Nucleic acid testing

At very early stage of the outbreak of COVID-19 pandemic (around early 2020), Chinese researchers sequenced genome information of the contagious virus, revealing its belongingness of the coronavirus (CoV) family, together with notorious SARS (severe acute respiratory syndrome) and MERS (middle East Respiratory Syndrome) [28]. Based on this recognition, the direct detection of SARS-CoV-2 by analyzing its RNA is then viable. To date, reverse transcriptase-polymerase chain reaction (RT-PCR) is mostly used in medically advanced countries and regions for surveilling the SARS-CoV-2 worldwide, considered as the “gold standard” technique to combat coronavirus disease. Basically, the viral RNA is collected in nasopharyngeal swab samples, then sent to RT-PCR for subsequent genome translation (to its complementary DNA) and amplification, for getting the sequenced results with high sensitivity. However, running this instrument is limited by time-consuming processes, low extraction efficiency, probable false positives (caused by contamination) and high costs [29–32]. However, community hospitals outside metropolitan cities and in many underdeveloped countries are consequently more difficult to cope with the pandemic stress. To this regard, nanomaterials especially the nanometals-based biosensing for SARS-CoV-2 detection is considerably on the rise owing to its precision, speed, robustness and affordability.

### Plasmonic gold nanoparticles and nano-islands

**Fig. 2:**
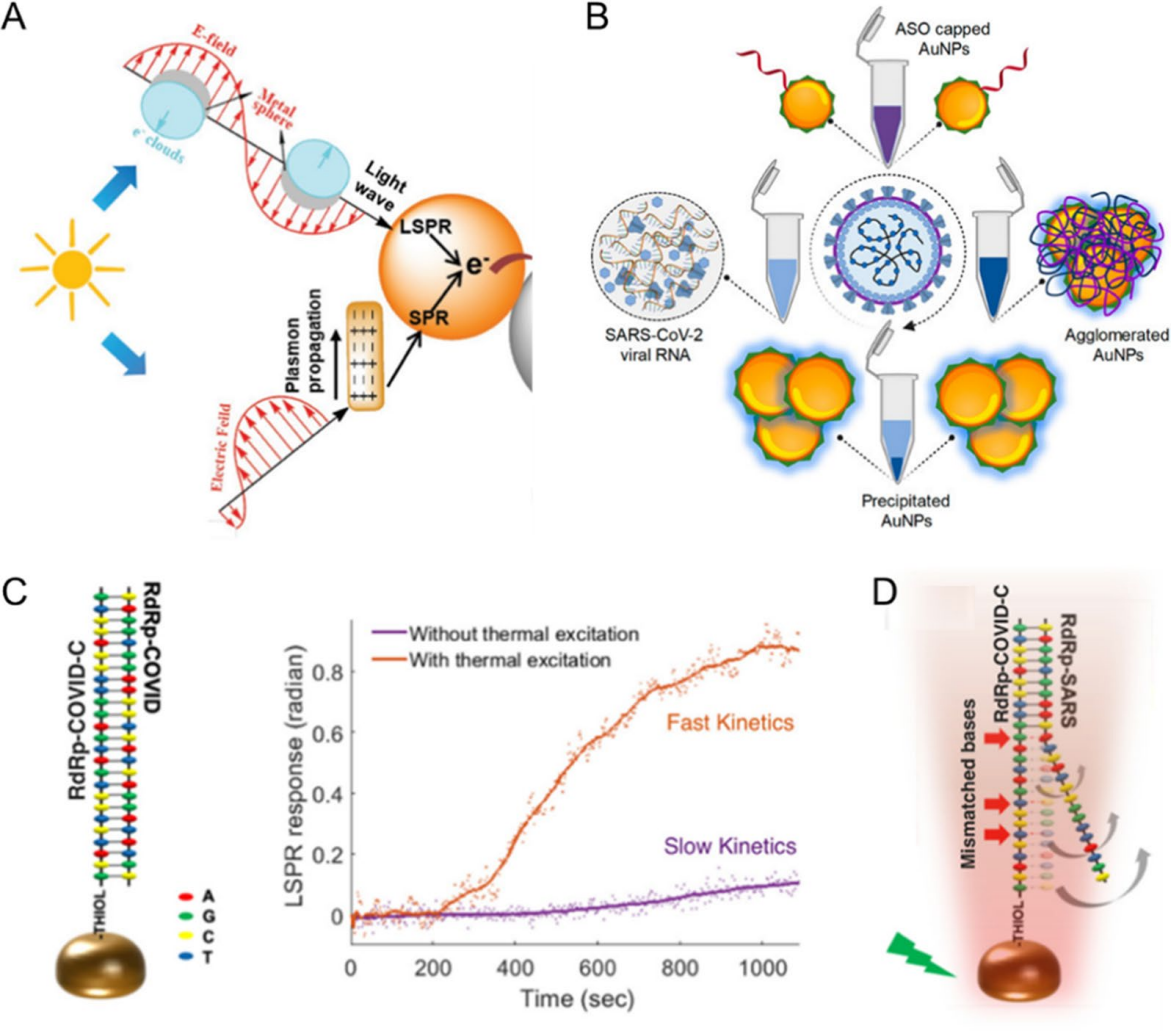
**A** Schematic illustration of the plasmon resonances for SPR and LSPR. Reprinted with permission [34]. Copyright 2019, Wiley–VCH. **B** Schematic illustration for antisense DNA-based AuNPs platform used in detecting the SARS-CoV-2 viral RNA. Reprinted with permission [46]. Copyright 2018, American Chemical Society. **C** Schematic illustration showing the two complementary oligonucleotides hybridized with each other, and their real-time interaction with or without the PPT treatment. **D** Schematic illustration showing the mismatched part detached from the cDNA strand. **C** and **D** are reprinted with permission [47]. Copyright 2020, American Chemical Society

Noble metals such as gold and silver can strongly interact with light, since the conduction electrons on their surface are highly free and undergo a collective oscillation at specific excitation wavelength. This oscillation is known as the surface plasmon resonance (SPR), which enables the light adsorption and scattering intensities of noble metals to be higher than identically sized non-plasmonic counterparts (Fig. 2A, the upper panel) [33, 34]. For metallic nanoparticles, when their sizes are smaller than incident wavelength, the light wave could be further trapped within nanoparticles to produce localized surface plasmon resonance (LSPR) (Fig. 2A, the lower panel). The localized electromagnetic field can be extremely enhanced [35], therefore noble metal nanostructured materials such as the gold nanoparticles have fascinated a lot attention and been actively used in developing sensitive and straightforward biosensors. [36–40]

To detect the SARS-CoV-2 related nucleic acids by gold nanoparticles (AuNPs), attaching thiol-modified complementary DNA (cDNA) sequences on nanogold surface is a well-accepted strategy [41–45]. When the targeted viral RNA is captured by their cDNA, the dispersion of AuNPs will change accordingly, resulting in shifted light adsorption and detectable output signals. For example, to sense N-gene of the SARS-CoV-2, Moitra et al. grafted a SH-modified antisense DNA on AuNPs (~ 55 nm), and observed color changes from purple to blue (red shift of 40 nm characterized by UV–vis) only in the presence of the target viral RNA (Fig. 2B) [46]. To further treat the hybridized RNA–DNA bridged nanoparticles by ribonuclease H (RNase H), the hybridized RNA was cleaved, leading to a visually detectable precipitate. The limit of detection was 0.18 ng/μL in this work. Compared to the time-consuming quantitative PCR with an assay time of 1–2 h, this platform measured no more than 10 min.

Aside to gold nanoparticles, two-dimensional gold nano-islands (AuNIs) were also exploited for viral nucleic acids detection, in which the plasmonic photothermal (PPT) effect and LSPR sensing transduction were combined. When illuminated the cDNA-modified AuNIs at their plasmonic resonance frequency of 532.2 nm, the localized PPT heat can help increase temperature on nanogold surface from 21.47 ℃ (room temperature) to 41.08 ℃ (Fig. 2C), therefore the mismatched viral sequence dissociated while only the right one can bind, resulting in more accurate discrimination between two similar viral genes (Fig. 2D) [47]. 0.22 pM viral sequence can be sensitively detected by the PPT-enhanced platforms.

Due to the LSPR effect of the AuNPs, they are also quite effective in quenching fluorescence through FRET (i.e. fluorescence resonance energy transfer), or enhancing through nanoparticle surface energy transfer [48]. For example, Hao et al. designed a platform combining oligonucleotide-tagged BaGdF_5_:Yb/Er upconversion nanoparticles (UCNPs) and Ebola virus sequence-attached AuNPs, in which the luminescence resonance energy transfer happened between them due to the spectral overlap of UCNP luminescence and AuNP adsorption [49]. Nevertheless, as far as we know that such energy transfer has not been utilized in developing nanogold-based sensors for detecting the viral RNA inside SARS-CoV-2.

Also, interestingly, Alafeef et al. reported a novel hafnium nanoparticle (HfNP)-based detecting platform for the RNA in SARS-CoV-2. They observed that the comparative band gap was reduced more for HfNPs than that for the AuNPs, contributing to a greater change in light absorbance and scattering. Their nanosensors impressively achieved a limit of detection of 0.06 copy/liter, i.e., 0.09 yM.

### CRISPR-mediated gold nanoparticles

**Fig. 3:**
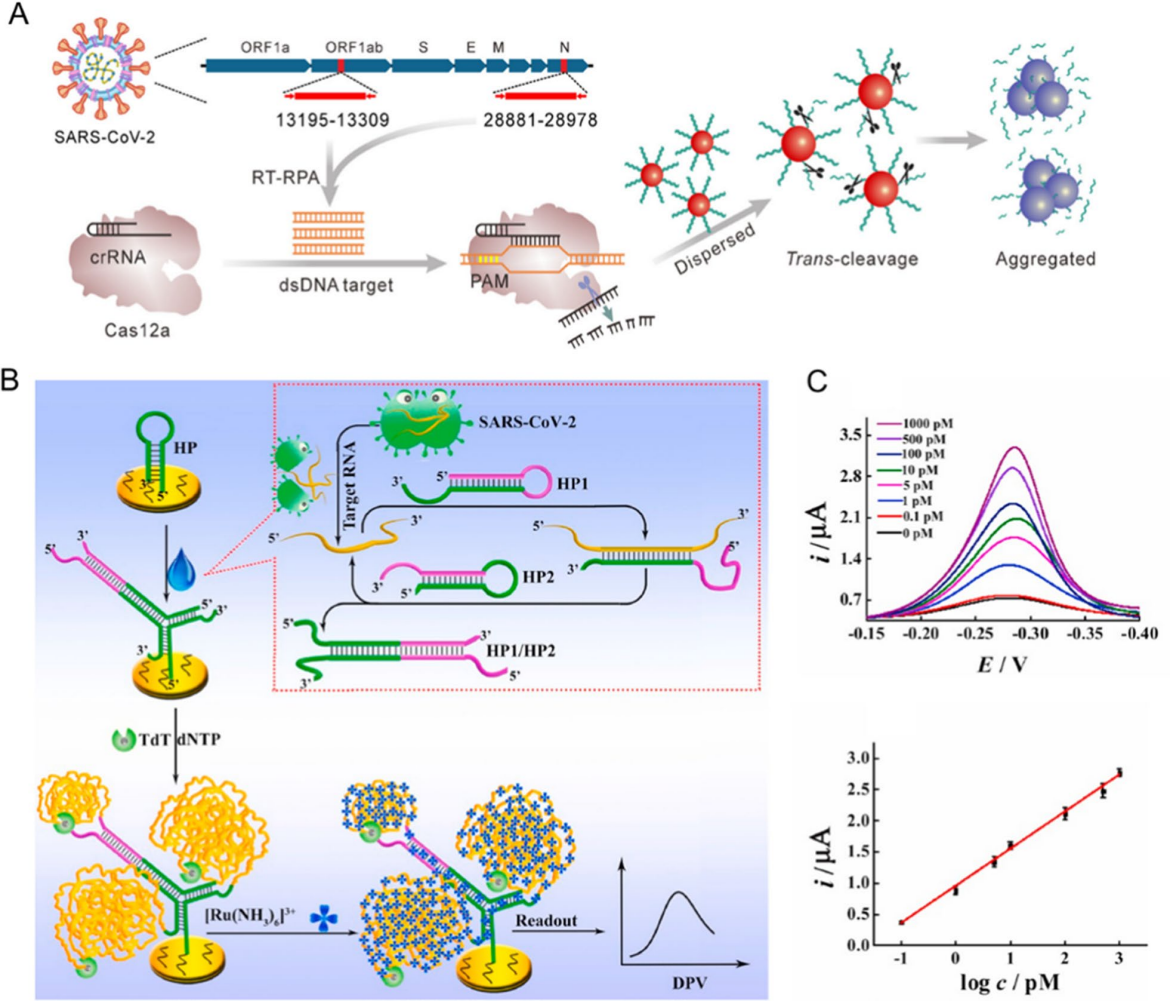
**A** Schematic illustration for RT-RPA-coupled Cas12a in binding and cutting the viral RNA, to generate visible color shift by modified AuNPs. Reprinted with permission [53]. Copyright 2021, American Chemical Society. **B** Schematic illustration for the CHA-combined electrochemical sensor for sensing the viral RNA. **C** Target RNA concentration-dependent response of the sensor, and the linear relationship between target RNA concentration and the current. **B** and **C** are reprinted with permission [66]. Copyright 2021, Elsevier Ltd

The AuNPs-based colorimetry is convenient and advantageous in naked-eye detection, as a consequence, it becomes highly attractive in hospital laboratory suffering from low instrument accessibility for screening. However, this traditional method is still constrained by low sensitivity and poor performance in determining ultralow level of nucleic acids, thus enhanced colorimetry is highly preferred. In the field of detecting SARS-CoV-2 RNA, clustered regularly interspaced short palindromic repeats (CRISPR)/Cas system, a cutting-edge tool for editing genomes [50–52], has been actively explored and combined into existing colorimetry for enhanced results. The CRISPR/Cas system uses RNA-guided nucleases to cut apart targeting genetic DNA or RNA, through Cas9, Cas12a and Cas13 protein, etc. Based on this understanding, Su and coworkers, for example, designed a metallic particles-mediated signal amplification strategy for SARS-CoV-2 detection. First, the virus genome was amplified through a reverse transcription recombinase polymerase amplification (RT-RPA), and then the obtained abundant dsDNA will specifically recognize and active Cas12a, so as to exhibit nonspecific trans-cleavage activity. Due to the trans-cleavage activity, the DNA conjugated AuNPs, as a result, were no longer stable and aggregated showing the color changes from red to purple (Fig. 3A) [53]. The sensitivity of this combined strategy reached 1 copy of viral genome sequence in per test. Likewise, Le and coworkers also incorporated the Cas12a system into hairpin DNA-functionalized AuNPs, in which the gRNA can recognize the amplicons of the N gene and E gene of SARS-CoV-2 to initiate the Cas12a nuclease *trans*-cleaving hairpin loop, resulting in AuNPs aggregation and color shift [54]. The authors tested 54 clinical respiratory swab samples by this colorimetric assay, generating 92.6% sensitivity and 100% specificity.

In addition to the above-mentioned Cas12a, another nuclease Cas13 that can chop up RNA in a nonspecific RNase manner has also been harnessed to enhance the calorimetry. Uttamapinant et al. isothermally amplified viral DNA sequence by RT-RPA, and converted them to RNA by T7 transcription, enabling the Cas13-crRNA complex to be further initiated to cleave the target RNA. They combined this to a colorimetric lateral-flow strip for visualized detection, and observed 100% specific and 97% sensitive readout in 154 nasopharyngeal and throat swab samples collected from a Thailand hospital. [55]

### Nanometals for electrochemical sensing

On the other hand, electrochemical biosensors are another alternative for sensitively analyzing the SARS-CoV-2 RNA [56–59]. It can monitor the COVID-19 in clinical diagnosis [60, 61], and also have the advantages of portability, miniaturization and low cost [62, 63]. Normally, the electrochemical biosensors contain three electrode cell configuration; a working electrode modified by cDNA or antibodies for recognizing the virus, a counter one and the other reference one. Modifying the electrode by nano metals or carbons may further enhance the sensitivity due to the increased surface area [64]. For instance, Singh et al. created a miniaturized electrochemical sensor on AuNPs-deposited titanium working electrode. Modifying the AuNPs by thiol-cDNA allow the sensor to distinguish a few piece of viral RNA in SRAR-CoV-2 (e.g. E protein genes). [65]

Recently, Li and coworkers further combined the signal amplification units of catalytic hairpin assembly (CHA) into electrochemical sensors for enhanced sensitivity [66]. Initially, two complementary DNA sequences were separately locked in metastable hairpin structures to inhibit their hybridization (named HP1 and HP2, in the upper panel of Fig. 3B). Then, addition of viral RNA will open up the HP2 and promote the interaction between HP1 and HP2. The combination of HP1/HP2 will specifically recognize HP on electrode surface and form a Y-shaped structure on electrode. Through adding terminal deoxynucleotidyl transferase (TdT), a template-free polymerase to catalyze the addition of nucleotides to the 3' ends of DNA, the strand was further extended (in the lower panel of Fig. 3B). Finally, adsorption of electroactive Ru(NH_3_)_6_^3+^ on such nucleic acid complex can generate sensitive signals, and allowed the detection of 0.1–1000 pM RNA with a limit of detection as low as 26 fM (Fig. 3C).

### Modified magnetic nanoparticles

Generally, the magnetic beads are used to extract and pre-concentrate target DNA or RNA strands from biofluids, such as whole blood, serum, sputum as well as urine [67–70]. After surface modification, specific nucleic acids and proteins can be captured by those magnetic beads, for further purification and analysis purposes. To address the COVID-19 issues, Yu et al. prepared poly(amino ester) with carboxyl groups (PC)-coated magnetic nanoparticles (pcMNPs), for binding the viral RNA through strong interaction between carboxyl groups and negative nucleic acids [71]. The extracted strands were than analyzed by RT-PCR. To further explore the usage of the magnetic nanoparticles during the pandemic, Lim and coworkers combined silver nanoparticle clusters with surface-enhanced Raman scattering (SERS)-based assay [72]. They modified the silver nanoparticle by three amine-tagged cDNA via (1-Ethyl-3-(3-dimethylaminopropyl)carbodiimide (EDC) coupling chemistry, for probing RdRp, E, and N gene regions of the SARS-CoV-2. Interestingly, they revealed that anisotropic Ag nanostructures such as Ag nanostars (AgNS) and Ag triangular nanoplates (AgTP) can enhance the sensitivity of binding RdRp genes of SARS-CoV-2 on SERS, with a very low limit of detection of 10 aM. The improvement was probably brought by higher degree of anisotropy and more edges in AgNS and AgTP. This work achieved highly sensitive RNA detection without any enzymatic amplification steps.

**Fig. 4:**
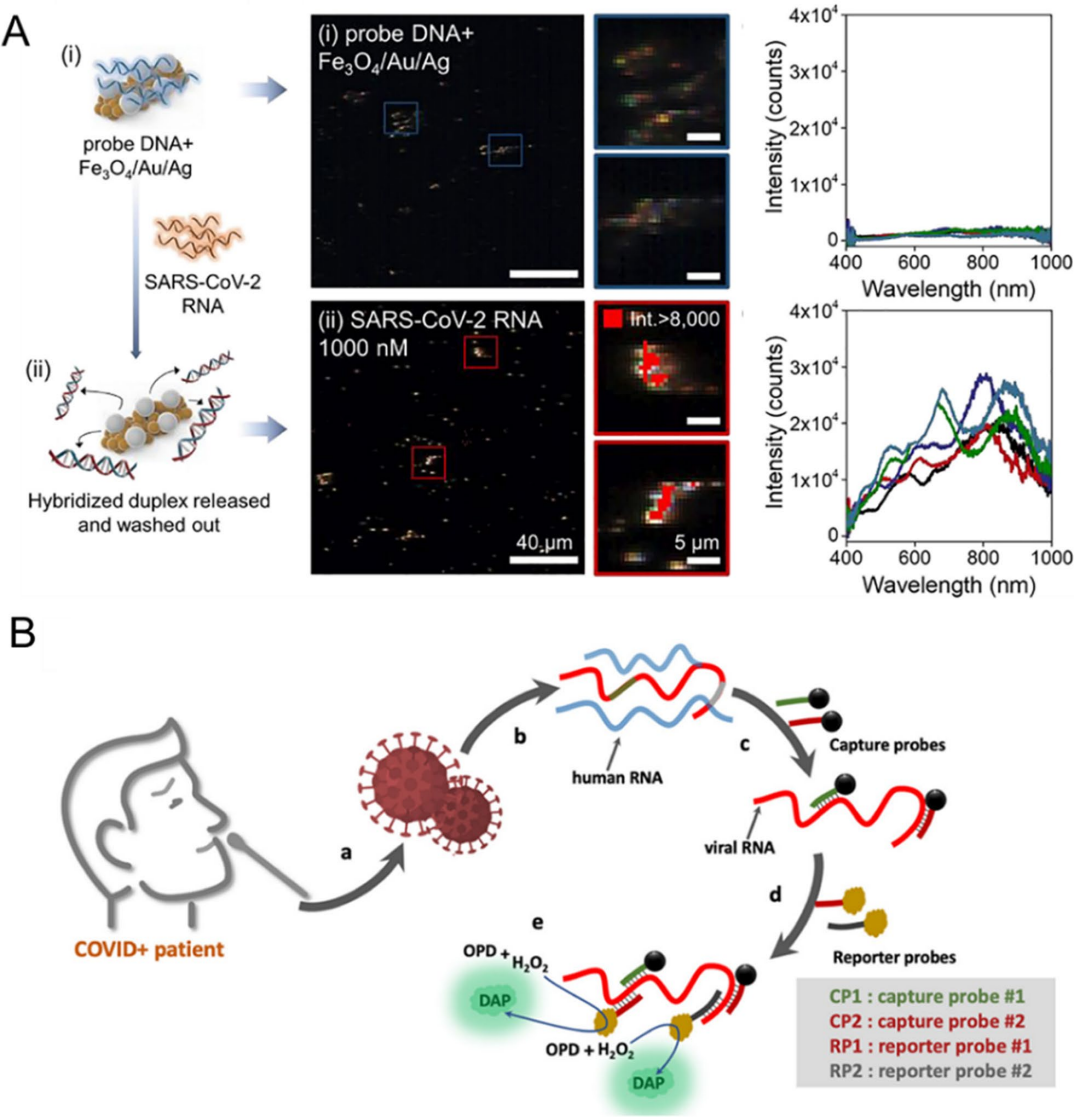
**A** Schematic of the hierarchically structured Fe_3_O_4_/Au/Ag NPs with or without the SARS-CoV-2 RNA. They were screened by HDFM respectively, in which the nanomaterials with viral RNA generated red overlaid spots indicating the notable signal. Reprinted with permission [73]. Copyright 2022, Elsevier Ltd. **B** Schematic illustration of the magnetic biosensor with HRP-terminated reporters for detecting the SARS-CoV-2 RNA. Reprinted with permission [74]. Copyright 2021, American Chemical Society

Moreover, Pumera and coworkers fabricated plasmonic-magnetic nanorobots consisting of Fe_3_O_4_ backbone and the outer surface of Ag for SARS-CoV-2 RNA detection. First, 10 nm Au seeds were synthesized on the Fe_3_O_4_ NPs and used to promote the nucleation and growth of Ag nanoparticles on Fe_3_O_4_ surface. Then, the hierarchically structured Fe_3_O_4_/Au/Ag NPs were assembled into rod-shaped microaggregates, which can be navigated and propelled under rotating magnetic field (Fig. 4A, the left panel) [73]. When attaching a DNA probe on Ag surface, SARS-CoV-2 RNA can be identified. Due to electrostatic changes, the formed duplex can be further released from nanorobots for RNA quantification. Through analyzing by hyperspectral dark-field microscopy (HDFM), it was visible that the Ag particles covered by ssDNA probe were dimmed at first, with quite low scattering intensities, while in the presence of viral RNA can release the duplex, then made the Ag particles to be shiny (Fig. 4A, the middle and right panels). This strategy generated a limit of detection to be 1.9 nM viral RNA. Finally, the authors designed an electrical readout platform for detecting the target RNA to validate its clinical applicability.

Additionally, Raouafi et al. developed another magnet fluorescent bioplatform by tethering two DNA probes on magnetic beads through biotin/streptavidin linkage, for specifically capturing the ORF1a and S genes of SARS-CoV-2. Upon RNA binding, it can be extracted from matrix (collected by nasopharyngeal swabs) under magnetic field. Then, two horseradish peroxidase (HRP)-contained sequence complementary to the S and N gene regions were added, which further allowed the oxidation of o-phenylenediamine (POD) to fluorescent products in the presence of H_2_O_2_, for RNA quantification purpose (Fig. 4B) [74]. Overall, the employment of magnetic nanoparticles enabled the SARS-CoV-2 RNA to be extracted and purified from complicated real samples [75], at the same time it can be combined with other sensing strategies for developing a variety of nanoplatforms.

### Lanthanides nanoprobes

**Fig. 5:**
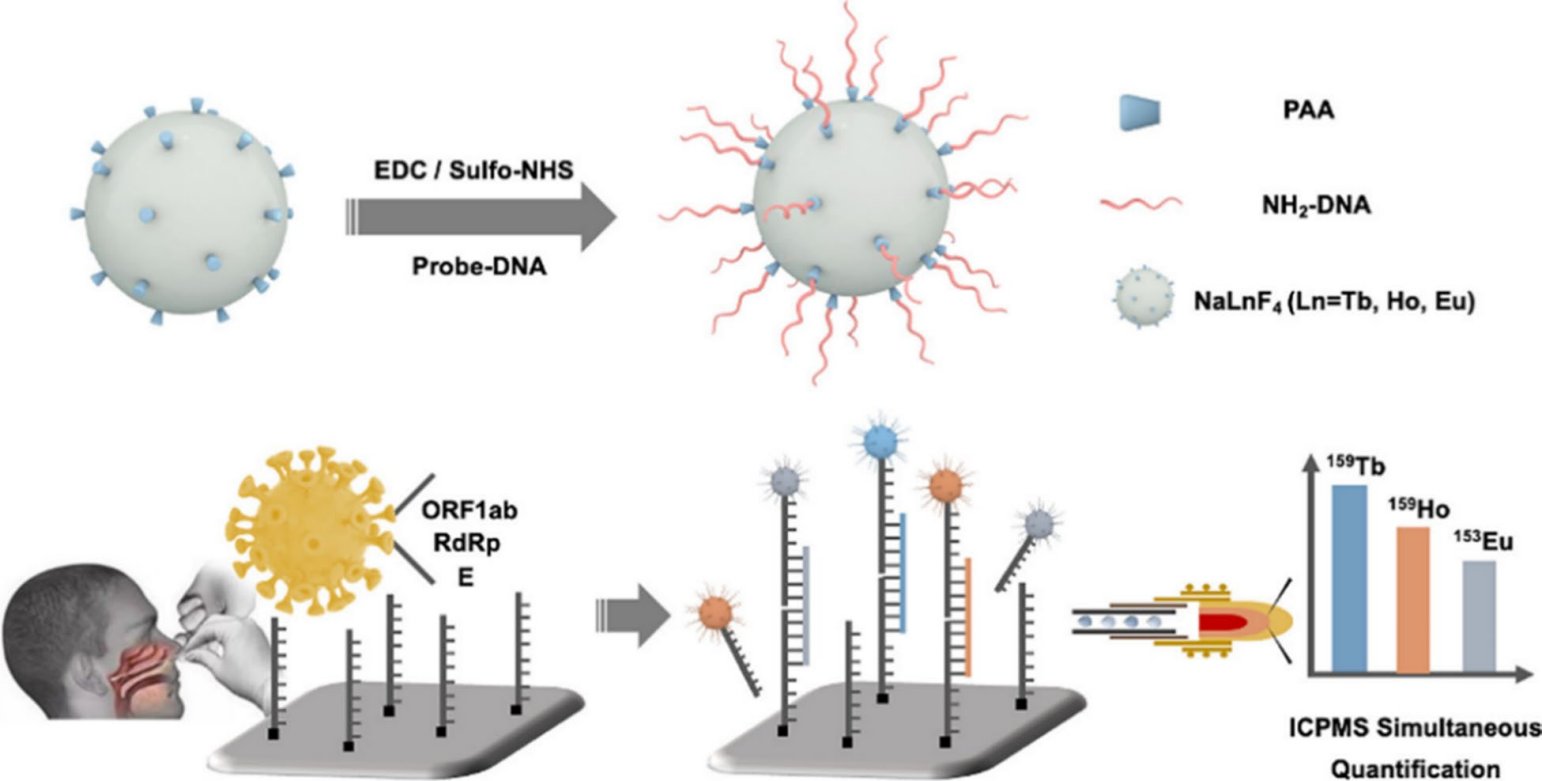
Schematic illustration of the probe DNA-modified NaLnF_4_ nanoparticles (coated by PAA) for detecting the RNA fragments ORF1ab, RdRp and E, by Tb, Ho and Eu-based platform, respectively. Reprinted with permission [77]. Copyright 2021, American Chemical Society

In addition to the aforementioned nanoplatforms for detecting viral nucleic acids, another choice is to harness transition metals of lanthanides (Ln). The nucleic acids can interact with Ln^3+^ via phosphate groups or nucleobases, in which the former provide electrostatic interactions while the later offer nitrogen containing ligands. Among nucleotides, the adenosine and guanosine phosphates can better associate with the Ln^3+^. In the case of employing lanthanides to sense DNA and RNA, they can serve as reporting groups, structural probes and catalytic cofactors due to their optical as well as catalytic properties [76]. To surveil the SARS-CoV-2, for example, Lv et al. prepared three different types of lanthanide nanoparticles (LnNPs) as independent signal reporter for simultaneously sensing three viral RNA sequences. They prepared the terbium (Tb), holmium (Ho) and europium (Eu)-based nanoparticles for ORF1ab gene, RdRp gene and E gene, respectively [77]. Through using polyacrylic acid (PAA) as a surfactant and a capping group on surface of LnNPs at the same time, the NH_2_-modified cDNA can be grafted to recognize the target RNA strands (Fig. 5). Then, the RNA-carried LnNP tags can be further digested and the Ln^3+^ was released for inductively coupled plasma mass spectrometry (ICP-MS) analysis.

## Protein testing

### Proteins on SARS-CoV-2 surface

In addition to direct detect the viral RNA, another line of thought is to analyze proteins on SARS-CoV-2 surface. As we mentioned above that the coronavirus’ proteins include S protein, E protein, M protein and N protein, among them, the S protein acting as the main target of neutralizing antibodies and the N protein being an excellent biomarker due to its strong immunogenicity, are used mostly for testing [78]. Although the current antigen tests provide rapid results, they still suffer from sensitivity issues [79]. To realize a more sensitive and faster antigen sensing, a lot of efforts have been made, especially the metal-based nanoplatforms.

#### Plasmonic gold nanoparticles

AuNPs are considered to be an important class of nanomaterials to design the colorimetric tools and electrochemical biosensors for not only the nucleic acids, but also the proteins [80]. During the outbreak of COVID-19, they are naturally used by many groups worldwide, mainly based on the principle of specific antigen–antibody immunoreaction. The specific immunoreaction leads to changes in the distance between AuNPs or the surface state of AuNPs, resulting in changes in the optical and electrochemical signals of AuNPs, therefore the presence of coronavirus in biofluids and clinical samples can be determined.

**Fig. 6:**
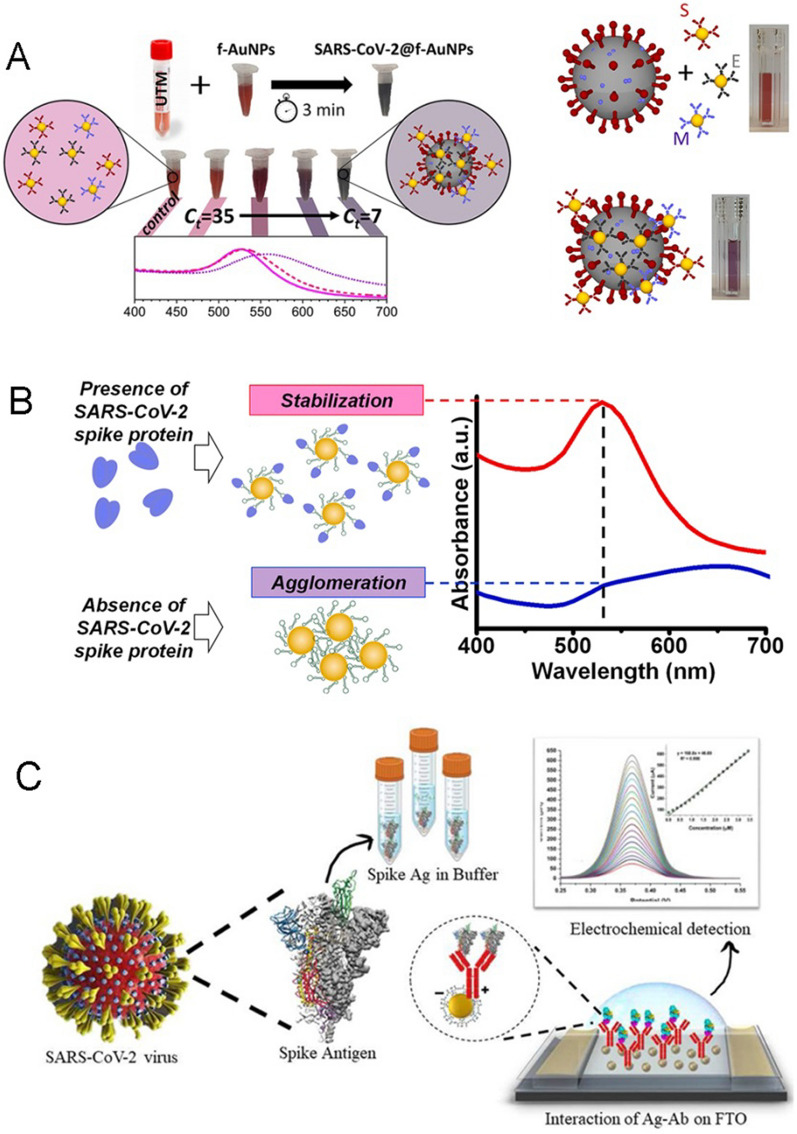
**A** Schematic illustration of the antibodies modified AuNPs used in detecting the surface proteins on SARS-CoV-2. Reprinted with permission [81]. Copyright 2020, American Chemical Society. **B** Schematic illustration of the aptamer-functionalized AuNPs for the detection of the surface proteins on SARS-CoV-2. Reprinted with permission [82]. Copyright 2021, Elsevier Ltd. **C** Schematic illustration of the electrochemical biosensor modified with AuNPs/SARS-CoV-2 antibodies for detection of spike protein. Reprinted with permission [83]. Copyright 2021, Elsevier Ltd

For example, Della Ventura et al. utilized functionalized AuNPs to direct detection of the virus (Fig. 6A) [81]. By using photochemical immobilization technique, antibodies targeting S protein, E protein, and M protein were decorated on AuNPs surface densely. When mixed with the solution containing viral particles, the interactions between AuNPs occurs, and the extinction spectrum of multiple viral-target AuNPs will be red-shifted within a few minutes. In such a system, the presence of SARS-CoV-2 virus in throat and nasal samples could be rapidly detected and the proposed nanobiosensor avoided the extraction and amplification of virus genome. The sensitivity and specificity were higher than 95%. Compared to the threshold cycle (Ct) of RT-PCR, the readout of the AuNPs-based nanobiosensors showed that the viral loads corresponding to Ct = 36.5 can be detected, indicating that this antigen sensing have the ability to detect very low viral load.

In addition, aptamers, defining as oligonucleotides that can specifically bind ligands, have been widely used in biosensing field as well owing to their high affinity, good specificity, routine synthetic process and modifiability [84, 85]. AuNP-aptamer conjugates are one of the most commonly used probes in analytical chemistry thus actively being used to detect the SARS-CoV-2-related proteins. Recently, AuNPs modified aptamers have been reported to have specific binding ability to S protein for stabilizing the colloidal golds [82]. As described in Fig. 6B, the presence of S protein and formation of aptamer-protein complex on AuNPs surface protected those particles from aggregation in a concentration-dependent manner. Based on this phenomenon, the collapse of LSPR peak was used to detect the SARS-CoV-2. The results showed that this AuNPs-aptamers nanoprobe exhibited the ability to detect 16 nM S proteins in phosphate buffer and 3540 genome copies/μl of inactivated SARS-CoV-2, indicating their excellent performance for SARS-CoV-2 detection.

Apart from colorimetric biosensors, AuNPs-based electrochemical biosensors have also attracted interests in the clinical diagnosis of COVID-19 due to their advantages of high sensitivity, low cost and portability. AuNPs enable direct electron transfer between biomolecules and electrode materials, thus allowing electrochemical sensing without electron transfer mediators [86]. Meanwhile, AuNPs can also serve as the signal amplifiers to increase the sensitivity. For example, Roberts et al. reported a fluorine doped tin oxide (FTO)-based electrochemical biosensor to detect S protein (Fig. 6C) [83]. By dropping AuNPs on FTO electrode and immobilizing the antibody (Ab) of S protein on AuNPs, a FTO/AuNPs/Ab based biosensor was fabricated, of which the limit of detection (LOD) in standard buffer and in saliva samples were calculated to be 0.63 fM and 120 fM, respectively. Moreover, this biosensor had a long storage shelf life and can be used as a sensitive diagnostic tool to detect S protein in clinical samples rapidly. Overall, the nanogold-based platforms are well-accepted and well-understood for detecting both the viral proteins and nucleic acids in COVID-19 surveilling.

#### Silver nanoparticles

Similar to AuNPs, silver nanoparticle (AgNPs) also have a high plasmonic effect, and the extinction coefficient of AgNPs is higher than that of AuNPs with the same average size [87]. Therefore, AgNPs are also widely used in sensor design. Although the surface of AgNPs is easy to oxidize, leading to their lower stability and popularity than AuNPs, some unique properties of AgNPs including strong antibacterial/antiviral activity and low-cost preparation method are attractive. Therefore, biosensors based on AgNPs for direct detection of viral protein have also been developed.

**Fig. 7:**
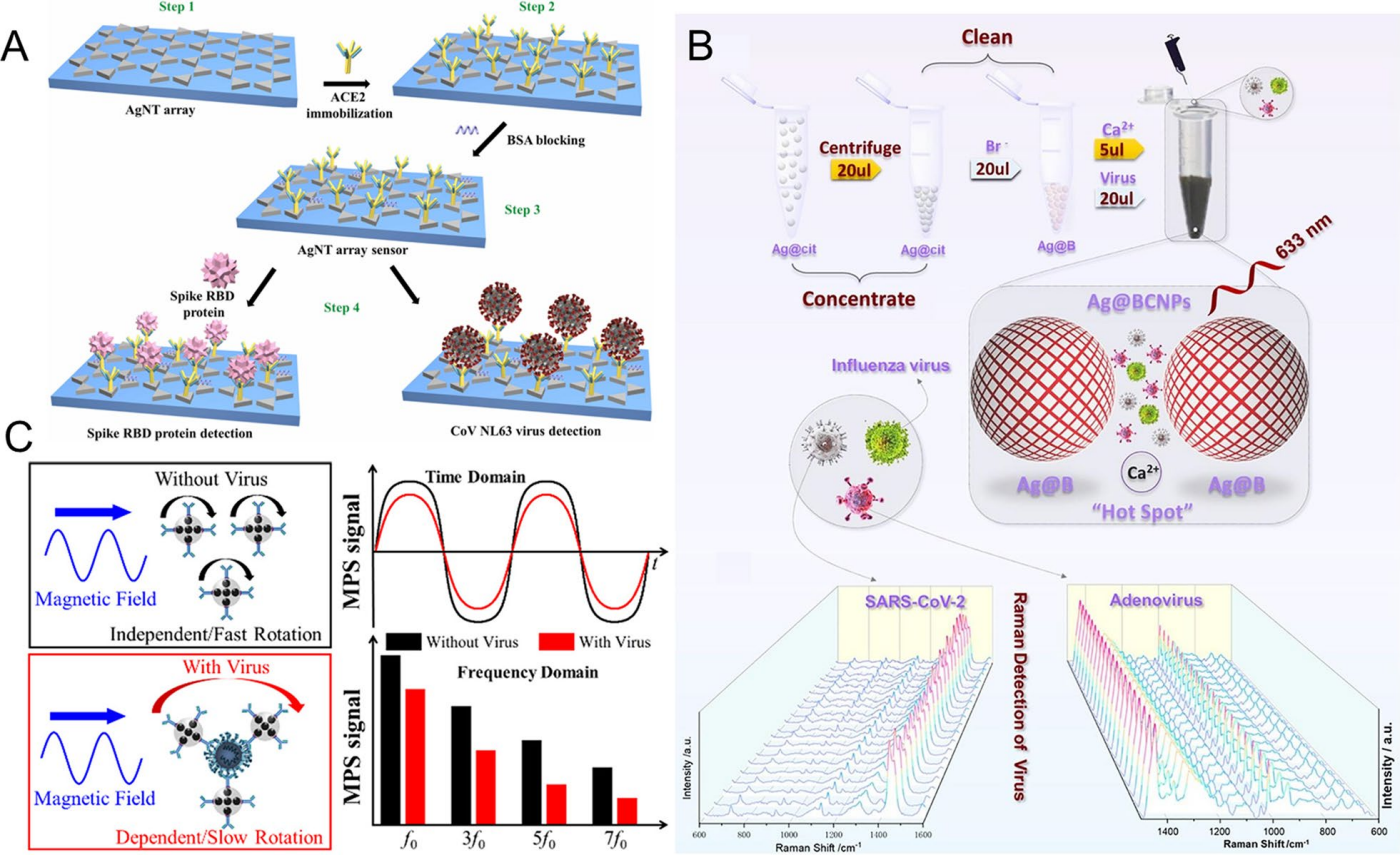
**A** Schematic diagram of the functionalized silver nanotriangle used as LSPR sensor for detecting spike protein and virus. Reprinted with permission [88]. Copyright 2022, Elsevier Ltd. **B** Schematic diagram of the AgNPs-based SERS platform for virus detection. Reprinted with permission [91]. Copyright 2022, Elsevier Ltd. **C** Schematic of the magnetic nanoprobes based on S protein antibody-functionalized magnetic nanoparticles for direct detection of SARS-CoV-2 virus. Reprinted with permission [93]. Copyright 2021, American Chemical Society

For example, a functionalized silver nanotriangle (AgNT) array was developed by Yang et al. as a localized surface plasmon resonance (LSPR) sensor for rapid coronavirus detection (Fig. 7A) [88]. To be specific, human angiotensin-converting enzyme 2 protein (ACE2), which is highly sensitive and specific to the receptor-binding domain of S protein (S_RBD_) and CoV NL63 virus, was modified on the surface of AgNT to endow AgNT with the ability to target viruses. Binding to the virus or S protein induced a linear shift of LSPR wavelength, so as to detect S protein or CoV NL63 virus. The results showed that the LOD for S_RBD_ protein was 0.83 pM, and that for CoV NL63 in buffer and untreated saliva were 391 PFU/mL and 625 PFU/mL, respectively. The detection time was less than 20 min, demonstrating that this rapid AuNT-based sensor may hold great potential in COVID-19 diagnosis. Similarly, based on the LSPR characteristics of AgNPs, Bhalla et al. developed a molecular imprinting technology to specific recognize the S_RBD_, so as to realize the sensitive and rapid detection of novel coronavirus multiple variants [89].

Because the Raman scattering intensity can be increased by several orders of magnitude when using suitable substrates and enhancer, the application of surface enhanced Raman spectroscopy (SERS) technology in biological systems shows great advantages. AuNPs and AgNPs are the most effective enhancers in SERS because their surface plasmons are located in the visible region of the electromagnetic spectrum and overlap with the laser excitation wavelength commonly used in Raman spectrum [90]. Therefore, AuNPs and AgNPs-based SERS have been integrated into biosensing to detect biomolecules. Recently, the SARS-CoV-2 detection platform based on AgNPs-related SERS has been developed and showed excellent performance. For example, by introducing acetonitrile and calcium ions into the AgNPs reinforced substrates, Zhang et al. designed a detection SERS platform to realize the rapid detection of SARS-CoV-2 (Fig. 7B) [91]. In this strategy, acetonitrile was used to significantly enhance the stability of AgNPs and amplify the calcium-induced AgNPs hot spots, thus realized highly sensitive SERS signals. The detectable concentration of SARS-CoV-2, H1N1 influenza virus and Human Adenovirus 3 can reach to 100 copies/test with satisfactory reproduction and signal-to-noise ratio.

#### Functionalized magnetic nanoparticles

Moreover, magnetic nanoparticles (MNPs), such as iron, cobalt, nickel and their oxide, have also been greatly explored for sensing the viral proteins because of their size-changing magnetic properties, good biocompatibility and electromagnetic targeting. When used as magnetic biosensors, the MNPs can be grafted by antibodies, aptamers and other functional molecules to detect and separate bacteria, viruses, and other microorganisms, exhibiting low background noise and rapid analysis properties [92]. Therefore, MNPs-based biosensors are considered to be one of the most promising methods for monitoring the SARS-CoV-2.

To detect spike proteins, Zhong et al. developed a homogeneous biosensor based on functional MNPs for direct detection of SARS-CoV-2 virus (Fig. 7C) [93]. The binding behavior between S protein antibody-functionalized MNPs and S proteins of the SARS-CoV-2 increased the hydrodynamic size of MNPs or form cross-linking structures. The change of MNPs’ hydrodynamic size significantly changed their Brownian relaxation time and dynamic magnetization in a time-varying magnetic field. Therefore, the shift of peak frequency in ac susceptibility (ACS) spectra and the change of the harmonics in magnetic particle spectroscopy (MPS) could be used to detect the binding behavior of SARS-COV-2. The LOD for the detection of mimic SARS-CoV-2 can reach to 0.084 nM (equivalent to 5.9 fmole), demonstrating the proposed MNPs-based biosensor holds great potential for rapid and sensitive diagnostics of SARS-CoV-2. Similarly, by monitoring the dynamic magnetic responses of MNPs and using the harmonics as a measure of the binding states, Wu et al. reported a MPS platform for the detection of S proteins and N proteins. Polyclonal antibodies anchored MNPs were used as nanoprobes and formed nanoparticle clusters when specially binding to SARS-CoV-2 S and N proteins, inducing the changes in higher harmonics [94]. The detection limits for S proteins and N proteins can reach to 1.56 nM (125 fmole) and 12.5 nM (1 pmol), respectively. This one-step, wash-free and MNPs-based MPS platform is intrinsically versatile and can be used for the detection of other disease biomarkers.

In addition to the MPS platform, the giant magnetoresistance (GMR) platform is also used in the design of MNPs-based biosensors. The spin interaction between MNPs and virus surface protein causes the change of resistance, leading to magnetization [95]. Besides, since blood, serum and other samples are non-magnetic, the magnetic signal detected in this way contains low background noise level. These MNPs-based sensors are expected to be designed into portable devices.

Furthermore, MNPs are also used to construct electrochemical biosensing. For example, based on antibody cocktail-conjugated MNPs, Durmas et al. developed an electrochemical immunosensor for the sensitive detection of SARS-CoV-2 virus and its variants [96]. After optimization, the LOD of this electrochemical immunosensor can reach to 0.53–0.75 ng/mL and its overall sensitivity, specificity can reach to 100%. The present MNPs-based biosensors have provided a range of versatile electrochemical platforms for the rapid and sensitive detection of virus and its variants.

### Secreted antibodies and cytokines

Aside from analyzing the surface protein on virus, secreted proteins during COVID-19 infection can also be monitored. Typically, IgM and IgG antibodies will be produced in patient’s serum a few days later upon the virus invasion [97]. From this regard, serological tests detecting IgM and IgG antibodies have been developed first as an indirect method for the COVID-19 diagnosing. Generally, IgM and specific antiviral IgG antibodies could be found 7 days and 10 days after symptom onset, respectively, while antibodies exist much longer in body fluids than viral RNA or antigens [98]. Therefore, serologic tests would be suitable for widespread screening of past infection and monitoring the disease progression, but not for early detection. So far, most serological tests for detecting SARS-CoV-2 related antibodies are based on traditional enzyme linked immunosorbent assay (ELISA) by employing recombinant coronavirus proteins: the S protein and the N protein [99]. However, antibody detection based on ELISA is quite time-consuming and costly. By comparison, metal-based nanoplatforms used for antibody detection are able to achieve fast, cheap and sensitive diagnosis of COVID-19.

Before discussing the specific detection methods, some concepts should be elaborated. As mentioned above, antibodies are a class of functional proteins, so the detection of antibodies is essentially the detection of proteins. However, compared with the detection of proteins on viral surface, the antibodies detection is different for monitoring the process of SARS-CoV-2 infection. First, the detection of surface proteins on virus is a direct way for the detection of virus, while targeting antibodies is an indirect method to indicate the existence or past existence of a specific virus. Second, to analyze viral surface proteins, the detection samples are collected from nasopharyngeal swabs, whereas that for antibody testing are obtained from blood; that means the sample pretreatment for biosensing is different. Third, the main purpose of determining proteins on viral surface is to identify infected people rapidly and economically to control the epidemic spread; yet the purpose for antibody detection is to widely screen the past infection and monitor the disease progression, rather than early detection. Therefore, we can see that those two protein-related detection methods do share some similarities, but also being complementary with each other in the diagnosis of COVID-19.

#### Gold nanoparticles

As we detailly discussed above that AuNPs extensively used for sensing viral nucleic acids and surface proteins, it is predictable that they are also utilized to trace those secreted IgM and IgG. For example, recently, a colorimetric strategy to detect SARS-CoV-2 IgG antibodies was studied by Lew et al. [100] By utilizing the AuNPs conjugated with short antigenic epitopes, the specific bivalent binding between IgG antibodies and epitope-functionalized AuNPs could be probed if the aggregation of AuNPs was triggered, resulting in distinct optical transition of AuNPs’ plasmon characteristics. The whole detection process can be completed within 30 min and the proposed biosensors showed nanomolar range of LODs for recognizing SARS-CoV-2 IgG antibodies.

#### Magnetic nanoparticles

**Fig. 8:**
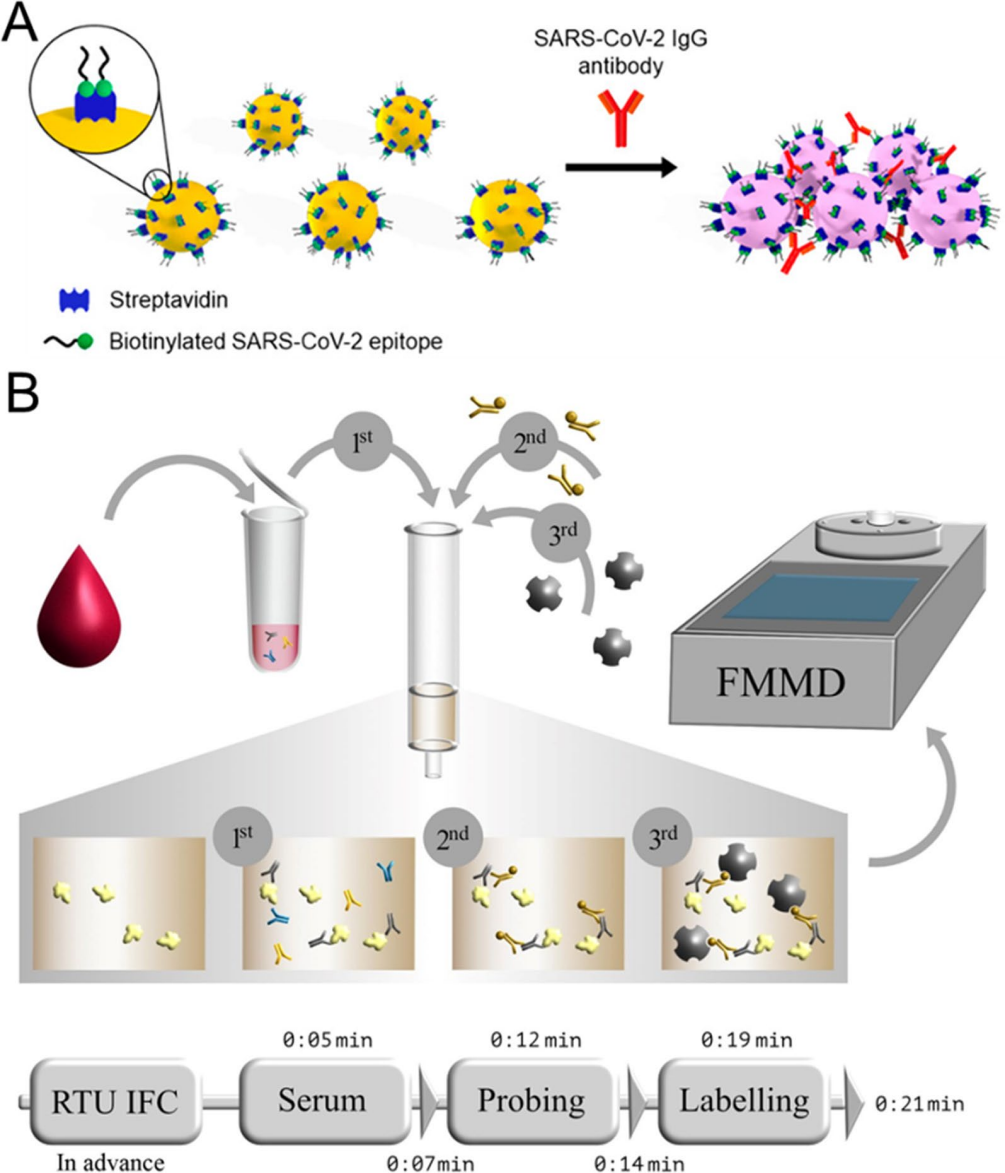
**A** Schematic representation of epitope-tagged AuNPs for the detection of SARS-CoV-2 IgG antibodies. Reprinted with permission [100]. Copyright 2021, American Chemical Society. **B** Schematic workflow of serological magnetic immunodetection for detection of SARS-CoV-2-specific antibodies in human serum. Reprinted with permission [101]. Copyright 2021, Frontiers Ltd

Since the outbreak of COVID-19, magnetic nanoparticles are also involved in the detection of COVID-19 diagnostic related antibodies. For example, Yadav et al. developed a new kind of gold-loaded nanoporous magnetic nanocube (Au@NPFe_2_O_3_ NC) as a dispersible capture and purifying agent for the electrochemical and naked eye detection of cancer-specific antibodies [102]. In a serological SARS-CoV-2 specific antibody detection assay, Pietschmann et al. first used immunofiltration columns to enrich human antibodies against SARS-CoV-2 (Fig. 8B) [101]. Then, an IgG-specific secondary antibody was used to bind the retained antibodies for further binding the IgG. Then, the IgG in biofluids was gradually enriched in column. The assay time is within 21 min with a sensitivity of 97% and a specificity of 92%, demonstrating the excellent performance of MNPs-based sensing for antibodies detection.

### Comparison of nucleic acid and protein testing

To surveillance the SARS-CoV-2, nucleic acid detection, antigen testing and antibody discrimination all have their advantages. For example, the nucleic acid testing is considered to be the most accurate one as the recognition of viral RNA relies on complementary base pairing and could be further enhanced by amplification strategies. In comparison, protein-related antigen testing is suitable for rapid screening of a larger number of infected individuals, by using the protein ligand (modified on nanobiosensors) that can specifically bind to antigen. Protein-related antibody testing also provides information including disease progression, past infection and vaccine-induced immunity of those patients. It can be said that these three main detection methods complement each other and together promote the strong detection ability of metal-based nanosensors.

These nucleic acid testing and protein testing based on metal nanoparticles do have something in common, such as the same means of signal output as we summarized by observing the color changes of gold nanoaggregates; meanwhile they are also different in many aspects. In this section, we would like to discuss more to compare these nanoplatforms (Table 2). Overall, metal nanoparticles that have been designed as colorimetric, electrochemical and magnetic biosensors all can be modified by oligonucleotide strands and proteins for neutralizing the target ligands in virus, so as to achieve the purpose of detection. However, the basic design principles of nucleic acid testing and protein testing are different. The former is mainly based on complementary base pairing rule (i.e. A pairs with T, and G pairs with C), while the later mainly depends on the specific antigen–antibody interaction. Such different design principles correspond to different ligand modification methods and different binding environments, which play an important role in the construction of biosensors. For example, for gold nanoplatform, the simplest and most effective method for nucleic acid modification is to conjugate the thiol group-containing nucleic acid to the surface of AuNPs via strong Au-thiol (Au–S) bonds. For the modification of proteins for binding viral antigens and antibodies, the modification methods depend on the properties of the protein, including non-covalent modifications such as electrostatic and hydrophobic interactions, and covalent modifications containing the use of thiol derivatives, bifunctional connectors and streptavidin–biotin. For magnetic nanoplatforms, its surface shell, such as polymer shell and Au shell, can be used to mobilize biomolecular probes. Similarly, direct conjugation of amino, carboxyl or thiol-modified nucleic acids to the surface of magnetic nanoparticles are also preferred for nucleic acid grafting, at the same time covalent and non-covalent modification strategies can be extended to protein attachment on their surface.

**Table 2:** The comparison of various metallic nanoplatforms for COVID-19 diagnostics

Platform	Target	Type of sensor	Sample type	LOD	Ref.
AuNPs	Viral RNA	Base pairing- mediated colorimetry	Oropharyngeal swab	0.18 ng/μL	[46]
	Viral RNA	CRISPR/Cas- mediated colorimetry	Upper respiratory specimens	10 pM	[53]
	Viral RNA	Electrochemical sensing	Upper respiratory specimens	26 fM	[66]
	Structural protein on virus	Antigen–antibody immunoreaction mediated colorimetry	Throat and nasal samples	Ct36.5	[81]
	S protein on virus	Antigen-aptamer interaction mediated colorimetry	Throat and nasal samples	3540 copies/μL	[82]
	S protein on virus	Antigen–antibody mediated electrochemical biosensors	Saliva samples	120 fM	[86]
	IgG antibodies	Antigen–antibody mediated immunoreaction	human plasma	3.2 nM	[100]
AuNIs	Viral RNA	Base pairing- mediated colorimetry	Upper respiratory specimens	0.22 pM	[47]
AgNPs	Virus	Antigen–antibody immunoreaction SERS	Inactivated SARS-CoV-2	100 copies/test	[91]
Fe_3_O_4_ NPs	Viral RNA	SERS-mediated assay	Upper respiratory specimens	10 aM	[72]
Fe_3_O_4_@Ag NPs	Viral RNA	Electronic readout signal	Upper respiratory specimens	1.9 nM	[73]
Magnet NPs	S protein on Virus	Binding-induced the change of magnetic signal	Mimic SARS-CoV-2	0.084 nM	[93]

Moreover, the pretreatment methods of different testing samples are different, since the viral RNA, antigen and antibody should be collected from various biofluids. For nucleic acid and antigen testing, nasal- and nasopharyngeal-swab samples are collected and placed in viral transport media, followed by mixing before testing. Differently, nucleic acid testing further requires an essential pretreatment step is to extract RNA with RNA extraction reagent, while for antigen detection, the swab eluted samples could be directly used without further treatment, contributing to faster and more convenient SARS-CoV-2 surveillance in real applications. Furthermore, to analyze the antibody secreted by patients, blood of patients is often collected. Followed by subsequent centrifugation as well as Triton X-100 treatment, the plasma could be separated and then diluted to 10%(v/v) for screening the past infection of target populations.

## Point-of-care test and others

Point-of-care testing (POCT) is a type of in vitro diagnostics (IVDs) that allows the medical diagnostic carried out at bedside [103]. In the world health organization (WHO) latest open Emergency Use Listing Procedure (EUL) for IVD, SARS-CoV-2 antigen detection tests and nucleic acid detection tests based POCT are listed as high priority [104]. POCT possesses several critical features which are of great significance in the controlling of COVID-19 pandemic breakout and following prognosis surveillance [105]. First, low requirements of samples. Unlike serological tests, samples for POCT are usually blood or body fluids without further treatments such as centrifugation or purification. Second, fast results readout. A typical POCT could be finished in minutes including the whole workflow of sample collecting, testing and results reading. Last, labor, time-saving, and unlimited application scenarios. As the WHO recently declared the COVID-19 global emergency is over, rapid and easy-to-use POCT testing will play a vital role in the post-pandemic era. Because POCT can be finished at any place and time without sending samples to the professional laboratory and sample manipulation with trained technicians, it is quite useful in the large population screening process. To date, there are many POCT applications were developed for SARS-CoV-2 detection and a considerable number of POCT products have been launched into the market [106]. Metallic nanomaterials’ size effects, large surface area, magnetic and plasmonic features could contribute to the sample collection and signal amplification. To further improve the traditional POCT sensitivity and specificity, nanometals were widely used in novel SARS-CoV-2 POCT developments.

### Optical-based point-of-care tests

**Fig. 9:**
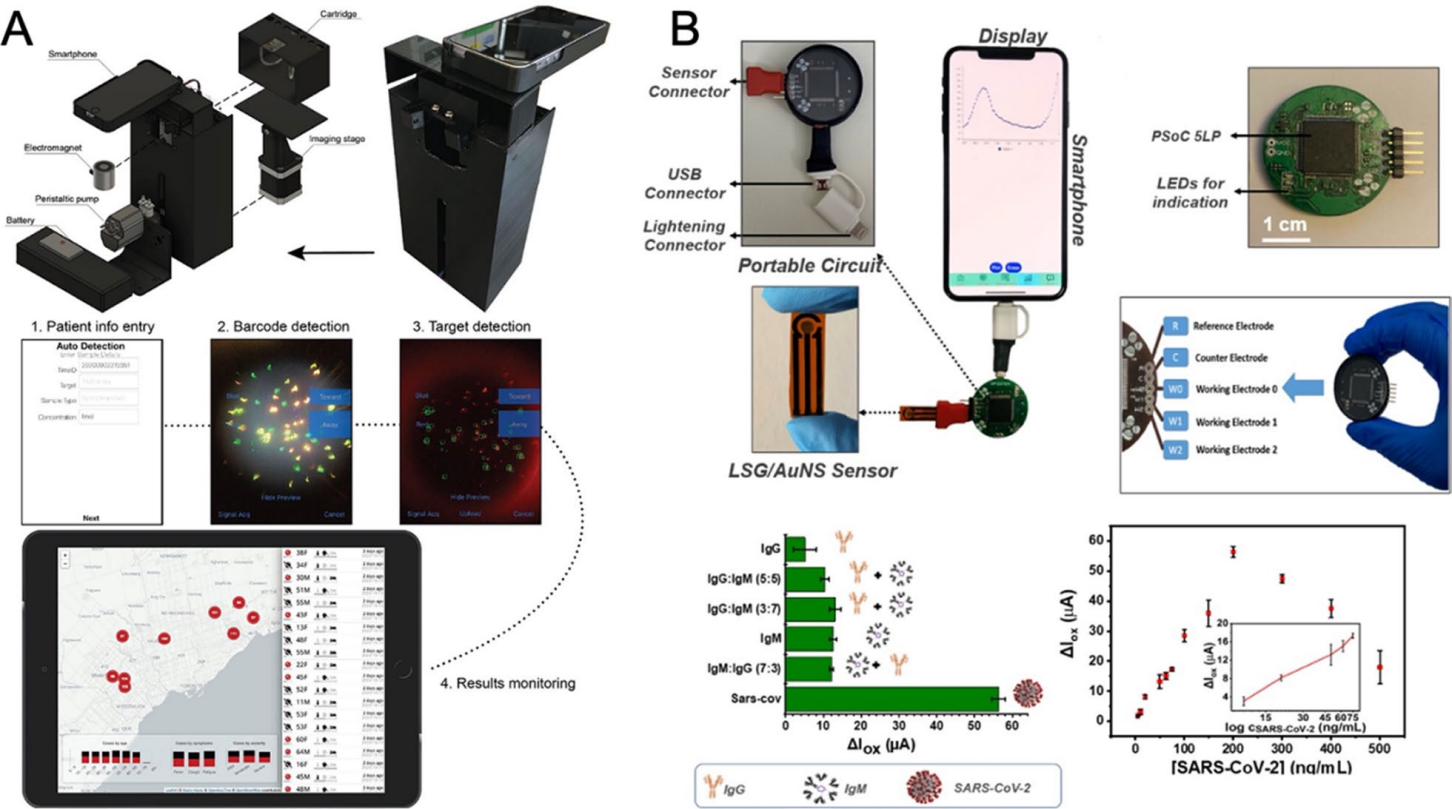
**A** Illustration of smartphone imaging device for quantum dot barcode-based COVID-19 immunoassay. [107] Reprint with permission, Copyright 2021, American Chemical Society. **B** Photo of the portable handmade COVID-19 POCT device. The device was connected to a smartphone via a USB-C connection to record the signal using a customized KAUSTat software. [108] Reprint with permission, Copyright 2021, American Chemical Society

Metallic nanomaterials possess unique optical properties such as plasmonic noble metal nanoparticles and fluorescent quantum dots which were widely used for biosensor development as mentioned above. In the category of POCT, these optical properties are extraordinarily attractive and the majority of reported SARS-CoV-2 POCT approaches were built upon metallic nanomaterials. For example, in a recent work developed by Zhang et al. a portable quantum dot smartphone device was fabricated for surveilling and tracking COVID-19 patients (Fig. 9A) [107]. Basically, they functionalized quantum dots with viral proteins for SARS-CoV-2 related antibodies capture. Next, sandwich structures were formed with fluorophore-conjugated secondary antibodies when the SARS-CoV-2 related antibodies presented. Finally, these quantum dot barcode immunoassay results were read out through a smartphone equipped with designed a databasing app. This work achieved real-time surveillance of patients infected with SARS-CoV-2 and was able to inform patients, physicians, and public health agencies instantaneously. Notably, in a head-to-head comparison with lateral flow assays, this POCT device achieved much higher clinical sensitivity (90% versus 34%) for SARS-CoV-2.

Taking advantage of the plasmonic nanoparticles’ fast thermocycling capability, it is possible to minimize the time and labor-consuming RT-qPCR pipeline into a portable device. In 2020, Cheong and coworkers developed a “nanoPCR” portable device that integrated reverse transcription, fast thermocycling, and in situ fluorescence detection to detect SARS-CoV-2 RNA in a very short time [109]. This nanoPCR device greatly shorten the time of RT-qPCR from hours to 17 min with multichannel detection ability at the same time. Besides, benchtop PCR's comparable limit of detection can be achieved which is 3.2 gene copies per microliter. Further clinical investigation contained 75 SARS-CoV-2 positive patients and an equal number of healthy volunteers demonstrated excellent and rapid detection of *N1*, *N2*, and *RPP30* gene targets with high accuracy (more than 99%).

As the first priority, easy-to-use is the most essential feature that should be considered in developing novel COVID-19 PCOTs. In 2021, Bokelmann et al. developed a POCT for SARS-CoV-2 bulk testing based on the colorimetric loop-mediated isothermal amplification (LAMP) [110]. With the help of magnetic beads, SARS-CoV-2 viral RNA in the samples of gargle lavage can be captured and enriched by hybridization capture-based RNA extraction. This magnetic capture assisted improved LAMP POCT effectively prevented false positives and realized single positive samples identification in pools with multiple negative samples. Meanwhile, the less requirements on centralized laboratory instruments and commercialized reagents using well controlled the cost per test (around 1 Euro per individual). In sum, metallic nanomaterials have played a crucial role in the development of optical-based COVID-19 POCT. Taking advantage of the metallic nanomaterials’ physicochemical properties, fluorescent, colorimetric and SERS POCT applications were developed with improved accuracy and sensitivity.

### Electrochemical-based point-of-care tests

To date, the most representative POCT application, glucometer, was developed based on an electrochemical biosensor and is indispensable for diabetics' daily glucose monitoring. Few months later after the breakout of COVID-19 pandemic, Alafeef et al. developed an electrochemical biosensor chip to quantitatively detect SARS-CoV-2 viral RNA. Through the integration of antisense oligonucleotides functionalized gold nanoparticles and graphene nanoplatform, this POCT chip could detect viral RNA in 5 min with high sensitivity and specificity. The performance of this POCT device was firstly evaluated on the Vero cells infected with SARS-CoV-2 virus. Further validation of this device's sensitivity and accuracy were carried out on the clinical samples collected from COVID-19 positive patients and healthy asymptomatic subjects who were prior confirmed by RT-PCR diagnostic kit. As the author claimed, this POCT device achieved high sensitivity of 231 copies per microliter and limit of detection of 6.9 copies per microliter without any further amplification. Most importantly, due to the feasibility of this electrochemical device, the ssDNA-conjugated AuNPs could be readily reprogramed to realize simultaneously target two separate regions of the same SARS-CoV-2 N gene which allowed the detection of genomic mutant SARS-CoV-2 virus.

With the help of gold nanoarchitecture, Beduk and coauthors successfully developed a miniaturized laser-scribed graphene (LSG)-based electrochemical biosensor and used for COVID-19 POCT (Fig. 9B) [108]. After the optimization and evaluation of this biosensor’s viral detection performance, it was finally connected with a smartphone via USB-C dock for the fabrication of portable handmade POCT device. The overall detection ability of this electrochemical POCT device was evaluated using the S-protein standard solution (5.0 − 500 ng/mL) with a detection limit of 2.9 ng/mL. Further clinical validation was carried out on 23 COVID-19 positive blood serum samples, and the results achieved the best agreement with the commercial RT-PCR test. This POCT device provided a promising alternative solution for the rapid detection of SARS-CoV-2. Integrating magnetic nanobeads with gold electrode-assisted electrochemical biosensor, a POCT device was developed by Li and coworkers [111]. This microfluidic chip can connect to a smartphone to realize a portable diagnosis of SARS-CoV-2 nucleocapsid protein with the LOD of 230 pg/mL in whole serum and 100 pg/mL in diluted serum. Since the electrochemistry-based POCT has been widely used for many years, integrating metallic nanomaterials with current developed electrochemical POCT is quite straightforward and can be readily achieved.

### Lateral flow immunoassay-based point-of-care tests

Lateral flow immunoassay (LFIA), also known as lateral flow immunochromatographic assay, is a rapid diagnostic technology based on immune colloidal gold technology that emerged in the 1990s. Basically, most of the LFIA builds upon a nitrocellulose membrane with prefixed specific antibodies in certain regions. Once the sample is dropped, it will move forward along the membrane and encounter the area where the antibody is immobilized, the corresponding antigen in the sample will specifically bind to the antibody. During this process, immune colloidal gold or immune enzyme staining responds and displays a certain color. Due to the flexibility of LFIAs, they are widely used in medical diagnostics with diverse application scenarios such as the most commonly used home pregnancy test. In terms of COVID-19, LFIA played a critical role in the fast screening of infected people before RT-PCR nucleic acid test. Especially in this post COVID-19 era, though antigen-based LFIA is generally less sensitive than RT-PCR or other nucleic acid amplification tests, LFIA serial testing (repeated test at least 48 h apart) are still recommended by the U.S. CDC for self-testing because the virus genetic materials might stay in the body up to 3 months resulting in a false-positive result of RT-PCR test [112]. To further improve the sensitivity and specificity, metallic nanomaterials were extensively used in the novel LFIA developments. [113–115]

**Fig. 10:**
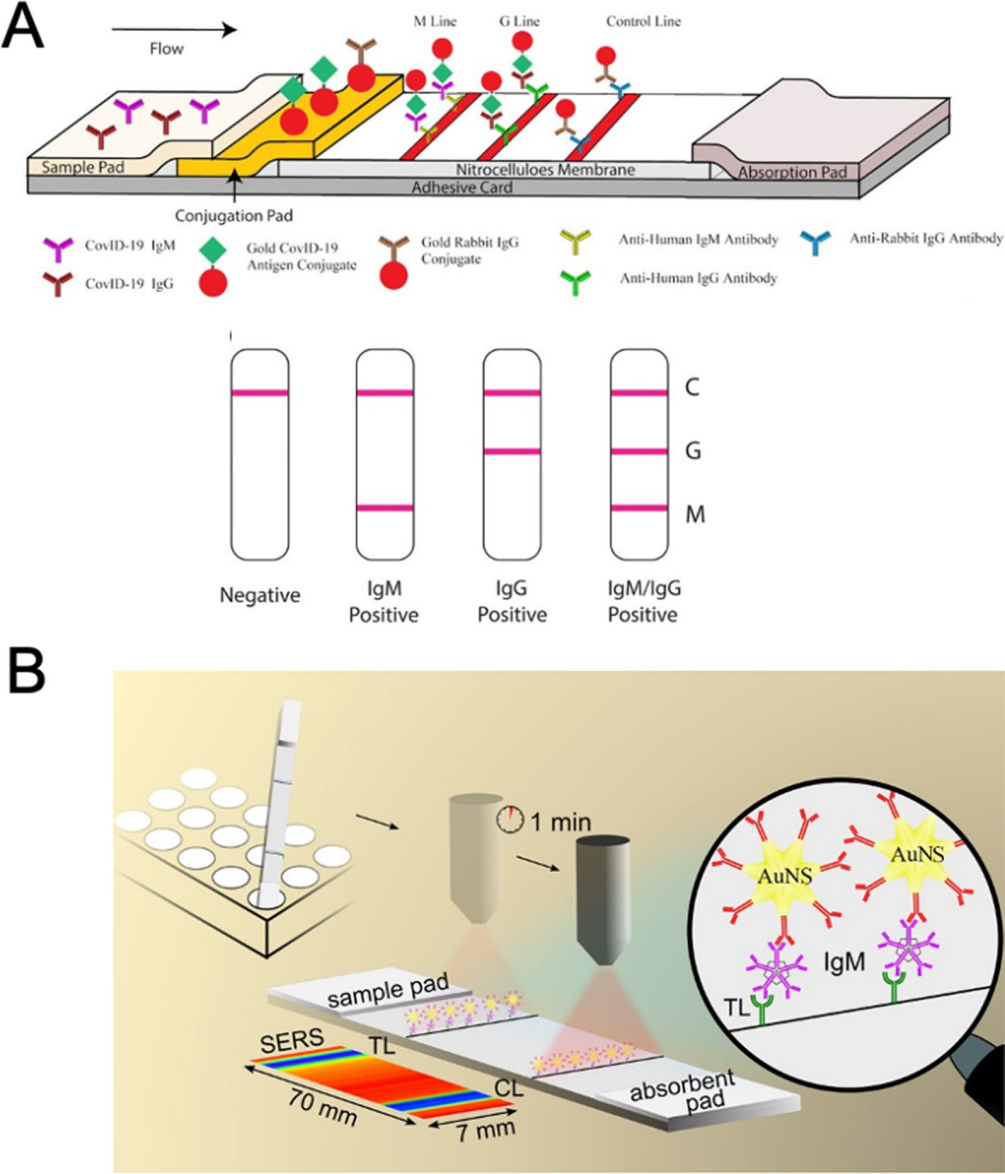
**A** Rapid IgM-IgG combined antibody Test for SARS-CoV-2 infection diagnosis [116]. **B** Schematics of the SERS-based POCT platform for SARS-CoV-2 specific IgM detection [117]. Reprint with permission, Copyright 2021, American Chemical Society

Developing paper-based immunoassays to detect SARS-CoV-2 and neutralizing antibodies is of great significance in the post COVID-19 era. The most common SARS-CoV-2 LFIA strip consists of a cellulose membrane with specific antigen or antibody immobilized and a binding pad adsorbed with colloidal gold labeling reagent (antibody or monoclonal antibody). When the sample is added, it moves forward via capillary action, reacts with the colloidal gold-labeled reagent. When moving to the area of fixed antigen or antibody, the sample and the gold-labeled reagent conjugates specifically bind to the fixed antigen or antibody, and gather on the detection zone, resulting in color development result which can be observed by naked eyes. Nanometals, such as AuNPs, have been used to prepare LFIA for the detection of IgM and IgG antibodies against SARS-CoV-2 antigens [116]. In a typical LFIA-based rapid-test strategy, antihuman IgM, IgG and antirabbit IgG antibodies (control sample) were separately modified in three different test lines on the nitrocellulose membrane (Fig. 10A). Then, AuNPs that functionalized the recombinant receptor binding domain of S protein was placed on the conjugation pad to capture antibodies. Therefore, the formation of the first and second red lines on the test strip indicated the presence of IgM and IgG antibodies. The whole process can be completed within 15 min and has a notable sensitivity (88.88%) and specificity (90.63%). In this regard, LFIA-based rapid-test strategy has great application prospects for rapid detection of COVID-19 infections.

In the 2021, Srivastav et al. developed a SERS-based LFIA for the rapid and sensitive detection of SARS-CoV2 specific antibodies [117]. Since the SERS uses the plasmonic property of gold nanoparticles, in addition to Raman signal readouts, it was possible to observe the test results at the control line and test line of the LFA strip with the naked eye as well. As shown in Fig. 10B, the gold nanoparticles were prelabeled with Raman reporter molecules. It could be used as conventional LFIA test strip and offered SERS signal readouts in the meantime. With the help of SERS amplification, this LFIA achieved more the 10 times increase in sensitivity compared with conventional LFIAs. Even though the test line was almost undetectable in naked eye readouts, SERS signals were still able to be detected with strong intensities. As an essential component of LFIA, fluorescence-based LFIA holds a large share of today’s POCT market as well. For instance, the fluorescence property of quantum dots [113], nanodiamonds [118], and lanthanide nanoparticles [119] was used to develop LFIA for SARS-CoV-2-Specific IgM/IgG and nucleocapsid protein detection.

Aside from as mentioned LFIAs which detect SARS-CoV2 related specific antibodies mostly, detection of other targets such as viral genes were also feasible. For example, Xiong et al. successfully developed a CRISPR/Cas9-mediated triple-line lateral flow assay (TL-LFA) for SARS-CoV-2 viral gene detection [113]. It was worth noting that this TL-LFA device utilized multiplex reverse transcription-recombinase polymerase amplification (RT-RPA) and realized simultaneously detection of two genes in a single strip test. Compared with conventional RT-qPCR methods, this POC platform was able to work in isothermal condition (37 ℃) without the need of temperature cycling allowing for the promotion in the different application scenarios. The whole assay procedure was limited within 1 h including viral RNA extraction which guaranteed a time-saving and sensitive detection with a LOD of 100 copies per reaction (25 mL). The performance of this CRISPR/Cas9-mediated TL-LFA method was also demonstrated in nasopharyngeal swab clinical samples showing comparable analytical specificity and sensitivity with the commercialized RT-PCR method.

## Metal based nanoplatforms for the post-COVID 19 era

It is now believed that the global effort in COVID-19 diagnosis and vaccination will bring the pandemic under control. Nevertheless, uncertainties remain about those constantly changing SARS-CoV2 variants, which may appear with seasonal epidemic peaks, be fueled by some immune-deficient individuals, or even pull us back into a new pandemic [120, 121]. Therefore, we critically discussed about how to cope with variants by having fast and accurate detection strategies on hand, and some relevant commercially available devices that we could use outside laboratory to closely surveil the SARS-CoV-2.

### Strategies for detecting SARS-CoV-2 variants

In the detection of SARS-CoV-2 variants, viral genome sequencing is the most accurate method [122]. However, genotyping methods based on single nucleotide polymorphisms often usually incur high economic and time costs, which is not conducive to epidemic prevention and control [123]. To cope with the problem of high cost, next generation sequencing and third generation sequencing are gradually used to detect SARS CoV-2 variants, but still cannot solve the time-consuming problem [124, 125]. Therefore, more other detection methods for detecting SARS-CoV-2 variants have been developed based on nucleic acid targeting and protein targeting.

RT-PCR is still the widely used technique for diagnosing SARS-CoV-2 infection, but the emergence of new variants can reduce the sensitivity of RT-PCR based diagnosis [126, 127]. To cope with this problem, some improving methods of PCR have been developed for identifying SARS-CoV-2 variants, including mutation-specific SARS-CoV-2 PCR [128], multiplex PCR [129, 130], loop-mediated isothermal amplification assay [131] and CRISPR-based methods [132]. For example, a mutation-specific RT-PCR technique based on VirSNiP mutation was designed. By deleting two amino acids (ΔE156/ΔF157) that cause the failure of S-gene target, Delta strains can be rapid screened [128]. This detection method exhibits high sensitivity and specificity, and is conceived as a rapid screening of Delta variant. However, since this detection method requires the deep understanding of specific mutations in various variants based on the results of gene sequencing, its applicability for possible future variants still needs continuous improvement. Furthermore, although those improved PCR-based detection technology has high sensitivity and specificity, these methodologies require competent laboratory personnel, complex instruments, and are time-consume [133]. On the other hand, CRISPR/Cas technology has been considered as an alternative to specifically identify mutations in the spike protein gene of SARS-CoV-2. When the CRISPR/Cas system encounters a matching sequence in the SARS-CoV-2 genome, it initiates a molecular reaction, resulting in a detectable signal. This signal can be visualized using various methods, such as fluorescent tags or colorimetric assays, allowing for quick and convenient identification of the viral genome including single-nucleotide mutations in the spike protein gene [134–136]. Since CRISPR-based methods can be performed isothermally, and do not require sophisticated equipment, they have promising potential for point-of-care testing [137, 138].

Fast antigen detection has always been used as an alternative option for detecting SARS-CoV-2. Although this detection is less sensitive than RT-PCR, it exhibits the advantage of instrument-free, short turnaround times and low-cost [139]. In the detection of SARS-CoV-2 variants, fast antigen detection plays an increasingly important role due to these variants, including Alpha, Beta, Gamma, Delta and Omicron, exhibit increased transmissibility and morbidity [140]. Furthermore, another advantage of rapid antigen detection is that it is still suitable for the detection of COVID-19 variants with the currently commercially available COVID-19 antigen sealing tube test strip [141]. For example, the globally popular colloidal gold test strip was investigated to verify its sensitivity and specificity for the diagnosis of the SARS-CoV-2 variants [141]. The detection results of 584 symptomatic and asymptomatic participants aged 0–90 years showed that the sensitivity of this test strip to Delta/Kappa variants L452R and E484Q S gene mutations was 96.97%, and the sensitivity to Omicron variant N501Y S gene mutations was 90.80%. This rapid antigen detection has a certain sensitivity to the detection of the widely spread Omicron variant, which can be used for self-inspection and POCT at home, thus strengthening the community management of COVID-19 [141]. Nevertheless, some reports on the diagnostic utility of antigen detection for variants have shown inconsistent results. Some studies showed the acceptable performance of antigen detection for variants [142, 143], but some studies verified a lower performance [144]. Therefore, the real performance of these assays should be further clarified to provide real information for decision makers.

### Metallic nanosensors for commercial uses

In the post-COVID 19 era, it is very important to develop commercially available devices that can be used outside laboratory to surveil the SARS-CoV-2. These commercially available antigen-detecting rapid diagnostic tests could increase the opportunity for early diagnosis of cases and accelerate clinical management decisions to reduce transmission [145]. Therefore, with the advent of the post epidemic era, a large number of COVID-19 test kits are needed. Recently, many COVID-19 antigen detection reagents have been approved for clinical use. Interestingly, metallic nanosensors play an important role in these approved kits. For example, 30 of the 40 COVID-19 antigen detection reagents approved by the National Medical Products Administration of China are based on the colloidal gold method due to its excellent performance. Other Metallic nanoparticles, such rare earth nanoparticles, magnetic nanoparticles, have also be used at the point-of-care [146]. These methods provide fast and affordable detections, but often encounter the problem of false positives. Therefore, it is still an urgent problem to improve the accuracy of these commercial kits.

### Metallic nanosensors compared to other nanoplatforms

In addition to metallic nanosensors, other nanoplatforms, including carbon-based nanoplatforms, polymer nanoplatforms etc., are also used for COVID-19 diagnostics in the post-COVID 19 era. These nanoplatforms can be also used with nasal swabs, throat swabs, serum, sputum samples or saliva samples. Although most of the current approved antigen diagnostic devices for COVID-19 are based on colloidal gold method due to the advantages of simple result-reading method and low cost, other nanoplatforms such as carbon nanomaterials still have their unique performance. For example, fluorescent carbon-based nanomaterials have the lower background and higher brightness, these carbon-based sensors have higher sensitivity and lower detection limit compared with colorimetric detection and can realize a sensitivity 10 times higher than colloidal gold method. However, fluoresce reading instruments are essential and necessary for signal reading. Nevertheless, the flashlight of smart phone may be the simplest solution to this problem. In addition to fluorescent carbon-based nanoplatforms, other carbon-based nanomaterials such as carbon nanotubes and graphene have also received a lot of attention due to their excellent electrical conductivity and optical property. However, some inherent drawbacks, such as the high price of carbon tubes and the difficulty to produce high-quality and purified graphene at an industrial scale, limit their widespread application in biosensing.

In other words, the wide use of metallic nanomaterials in the COVID-19 diagnosis accelerates the field of metallic nanomaterials-based bioanalysis. With advancements in nanotechnology, it is possible to create customized metallic nanomaterials with tailored properties that can be specifically designed for different types of biosensors. To date, metallic nanoparticles have been used for the detection of various biomarkers associated with diseases and provide high sensitivity and specificity, enabling the detection of these biomarkers at very low concentrations. Overall, the use of metallic nanomaterials in biosensing applications has vast potential for improving healthcare and advancing medical research. Ongoing research in this field will continue to push the boundaries of what is possible, leading to new and innovative technologies that can revolutionize the diagnosis and treatment of diseases.

## Outlook and conclusion

Accurate and rapid detection of SARS-CoV-2 infected people is essential to controlling the spread of COVID-19 pandemic. As mentioned by the WHO, asymptomatic carriers who are infected with SARS-CoV-2 but show little or no symptoms of the disease occupied a considerable proportion of the total infection numbers either in some regions or word widely. Therefore, it is difficult to identify the SARS-CoV-2 positive population by routine temperature monitoring or symptomatic observation. From this regard, the use of nucleic acid tests and protein or antibodies tests are important and should be more accurate. On the other hand, SARS-CoV-2 virus shows a fast-mutating nature, new variants of infection dominate the newly identified cases in months. These mutations in the SARS-CoV-2 virus will potentially impact diagnostic accuracy via several factors, including the sequence of the variant genetic information, the design of the diagnostic strategy, and the prevalence of the variant in different regions. Thus, more efforts are urgently needed to devote to the improvement of present diagnostic methods’ sensitivity and specificity, as well as the capability to deal with the challenges from rapid emerging SARS-CoV-2 variants. For example, tests with multiple targets mean detecting various sections of the viral genome, or different viral proteins at the same time. This strategy will improve the diagnostic accuracy to cope with the challenges posed by new variants of SARS-CoV-2.

Aside from the accuracy and sensitivity concern mentioned above, future developing trend of COVID-19 diagnosis and surveillance should pay more attention on other aspects. Firstly, the extreme high transmission ability of new SARS-CoV-2 variants makes COVID-19 diagnosis a part of our daily life. It is important to rational choose ideal diagnostic strategies in specific scenario. For example, tests at the airport should be accurate to control the spread of the pandemic, yet less accuracy is acceptable for medical staff who are frequently tested during daily checks. Second, most of the nanomaterials can be readily synthesized and functionalized at the bench side, scale-up of the production with strict quality control is challenging. Introducing the latest nanomaterials synthesis techniques and functionalization strategies to guarantee large-scale production and robust storage conditions is essential. Generally speaking, metallic nanomaterials are used for either signal amplification or simplifying and saving time in the COVID-19 diagnosis and surveillance. In this review, we systematically summarized recent progress of metallic nanomaterials-based SARS-CoV-2 diagnostic biosensors covered different targets and application scenarios. The introduction of metallic nanomaterials into these bioanalysis applications greatly improved the overall detection performance not only in the sensitivity and specificity but facilitated the test accessibility such as POCT devices. It is expected that using metal-based nanomaterials in the COVID-19 diagnosis and surveillance is an effective path to control the spread of this viral pandemic. With the broad employment of these novel diagnostical devices and the developments of vaccines or specific drugs, the COVID-19 pandemic will be end eventually as it transforms into an endemic disease.

## Data Availability

All data generated or analysed during this study are included in this published article.

## References

[1] Haldane V, Foo CD, Abdalla SM, Jung AS, Tan M, Wu SS, Chua A, Verma M, Shrestha P, Singh S, Perez T, Tan SM, Bartos M, Mabuchi S, Bonk M, McNab C, Werner GK, Panjabi R, Nordstrom A, Legido-Quigley H (2021). Health systems resilience in managing the COVID-19 pandemic: lessons from 28 countries. Nat Med.

[2] Verschuur J, Koks EE, Hall JW (2021). Observed impacts of the COVID-19 pandemic on global trade. Nat Hum Behav.

[3] Coronavirus disease (COVID-19) Weekly Epidemiological Update and Weekly Operational Update; World Health Organization. https://www.who.int/emergencies/diseases/novel-coronavirus-2019/situation-reports. Accessed 5 May 2022.

[4] Tregoning JS, Flight KE, Higham SL, Wang ZY, Pierce BE (2021). Progress of the COVID-19 vaccine effort: viruses, vaccines and variants versus efficacy, effectiveness and escape. Nat Rev Immunol.

[5] Teijaro JR, Farber DL (2021). COVID-19 vaccines: modes of immune activation and future challenges. Nat Rev Immunol.

[6] Udugama B, Kadhiresan P, Kozlowski HN, Malekjahani A, Osborne M, Li VYC, Chen H, Mubareka S, Gubbay JB, Chan WCW (2020). Diagnosing COVID-19: the disease and tools for detection. ACS Nano.

[7] Zhao ZL, Wang YL, Qiu LP, Fu T, Yang Y, Peng RZ, Guo MY, Mao LC, Chen CY, Zhao YL, Tan WH (2021). New insights from chemical biology: molecular basis of transmission, diagnosis, and therapy of SARS-CoV-2. CCS Chem.

[8] Harvey WT, Carabelli AM, Jackson B, Gupta RK, Thomson EC, Harrison EM, Ludden C, Reeve R, Rambaut A, Peacock SJ, Robertson DL, COVID-19 Genomics UK (COG-UK) Consortium (2021). SARS-CoV-2 variants, spike mutations and immune escape. Nat Rev Microbiol.

[9] Tao KM, Tzou PL, Nouhin J, Gupta RK, de Oliveira T, Pond SLK, Fera D, Shafer RW (2021). The biological and clinical significance of emerging SARS-CoV-2 variants. Nat Rev Genet.

[10] Kissler SM, Fauver JR, Mack C, Tai CG, Breban MI, Watkins AE, Samant RM, Anderson DJ, Metti J, Khullar G, Baits R, MacKay M, Salgado D, Baker T, Dudley JT, Mason CE, Ho DD, Grubaugh ND, Grad YH (2021). Viral dynamics of SARS-CoV-2 variants in vaccinated and unvaccinated persons. N Engl J Med.

[11] Twohig KA, Nyberg T, Zaidi A, Thelwall S, Sinnathamby MA, Aliabadi S, Seaman SR, Harris RJ, Hope R, Lopez-Bernal J, Gallagher E, Charlett A, De Angelis D, Presanis AM, Dabrera G, COVID-19 Genomics UK (COG-UK) Consortium (2022). Hospital admission and emergency care attendance risk for SARS-CoV-2 delta (B.1.617.2) compared with alpha (B.1.1.7) variants of concern: a cohort study. Lancet Infect Dis.

[12] Shiehzadegan S, Alaghemand N, Fox M, Venketaraman V (2021). Analysis of the delta variant B.1.6.17.2 COVID-19. Clin Practice.

[13] Tracking SARS-CoV-2 variants. World Health Organization. https://www.who.int/activities/tracking-SARS-CoV-2-variants. Accessed 31 May 2022.

[14] Khandia R, Singhal S, Alqahtani T, Kamal MA, El-Shall NA, Nainu F, Desingu PA, Dhama K (2022). Emergence of SARS-CoV-2 Omicron (B.1.1.529) variant, salient features, high global health concerns and strategies to counter it amid ongoing COVID-19 pandemic. Environ Res.

[15] Ji T, Liu Z, Wang G, Guo X, Akbar Khan S, Lai C, Chen H, Huang S, Xia S, Chen B, Jia H, Chen Y, Zhou Q (2020). Detection of COVID-19: a review of the current literature and future perspectives. Biosens Bioelectron.

[16] Fan HH, Lou FX, Fan JF, Li MC, Tong YG (2022). The emergence of powerful oral anti-COVID-19 drugs in the post-vaccine era. Lancet Microbe.

[17] Vandenberg O, Martiny D, Rochas O, van Belkum A, Kozlakidis Z (2021). Considerations for diagnostic COVID-19 tests. Nat Rev Microbiol.

[18] Mercer TR, Salit M (2021). Testing at scale during the COVID-19 pandemic. Nat Rev Genet.

[19] Ai T, Yang Z, Hou H, Zhan C, Chen C, Lv W, Tao Q, Sun Z, Xia L (2020). Correlation of chest CT and RT-PCR testing for coronavirus disease 2019 (COVID-19) in China: a report of 1014 cases. Radiology.

[20] Jin Z, Du X, Xu Y, Deng Y, Liu M, Zhao Y, Zhang B, Li X, Zhang L, Peng C, Duan Y, Yu J, Wang L, Yang K, Liu F, Jiang R, Yang X, You T, Liu X, Yang X, Bai F, Liu H, Liu X, Guddat LW, Xu W, Xiao G, Qin C, Shi Z, Jiang H, Rao Z, Yang H (2020). Structure of Mpro from SARS-CoV-2 and discovery of its inhibitors. Nature.

[21] Gordon DE, Jang GM, Bouhaddou M, Xu J, Obernier K, White KM, O’Meara MJ, Rezelj VV, Guo JZ, Swaney DL, Tummino TA, Hüttenhain R, Kaake RM, Richards AL, Tutuncuoglu B, Foussard H, Batra J, Haas K, Modak M, Kim M, Haas P, Polacco BJ, Braberg H, Fabius JM, Eckhardt M, Soucheray M, Bennett MJ, Cakir M, McGregor MJ, Li Q, Meyer B, Roesch F, Vallet T, Mac Kain A, Miorin L, Moreno E, Naing ZZC, Zhou Y, Peng S, Shi Y, Zhang Z, Shen W, Kirby IT, Melnyk JE, Chorba JS, Lou K, Dai SA, Barrio-Hernandez I, Memon D, Hernandez-Armenta C, Lyu J, Mathy CJP, Perica T, Pilla KB, Ganesan SJ, Saltzberg DJ, Rakesh R, Liu X, Rosenthal SB, Calviello L, Venkataramanan S, Liboy-Lugo J, Lin Y, Huang X-P, Liu Y, Wankowicz SA, Bohn M, Safari M, Ugur FS, Koh C, Savar NS, Tran QD, Shengjuler D, Fletcher SJ, O’Neal MC, Cai Y, Chang JCJ, Broadhurst DJ, Klippsten S, Sharp PP, Wenzell NA, Kuzuoglu-Ozturk D, Wang H-Y, Trenker R, Young JM, Cavero DA, Hiatt J, Roth TL, Rathore U, Subramanian A, Noack J, Hubert M, Stroud RM, Frankel AD, Rosenberg OS, Verba KA, Agard DA, Ott M, Emerman M, Jura N, von Zastrow M, Verdin E, Ashworth A, Schwartz O, d’Enfert C, Mukherjee S, Jacobson M, Malik HS, Fujimori DG, Ideker T, Craik CS, Floor SN, Fraser JS, Gross JD, Sali A, Roth BL, Ruggero D, Taunton J, Kortemme T, Beltrao P, Vignuzzi M, García-Sastre A, Shokat KM, Shoichet BK, Krogan NJ (2020). A SARS-CoV-2 protein interaction map reveals targets for drug repurposing. Nature.

[22] Wang W, Xu Y, Gao R, Lu R, Han K, Wu G, Tan W (2020). Detection of SARS-CoV-2 in different types of clinical specimens. JAMA.

[23] Tahamtan A, Ardebili A (2020). Real-time RT-PCR in COVID-19 detection: issues affecting the results. Expert Rev Mol Diagn.

[24] Chellasamy G, Arumugasamy SK, Govindaraju S, Yun K (2020). Analytical insights of COVID-19 pandemic. TrAC, Trends Anal Chem.

[25] Abid SA, Ahmed Muneer A, Al-Kadmy IMS, Sattar AA, Beshbishy AM, Batiha GE-S, Hetta HF (2021). Biosensors as a future diagnostic approach for COVID-19. Life Sci.

[26] Srivastava M, Srivastava N, Mishra P, Malhotra BD (2021). Prospects of nanomaterials-enabled biosensors for COVID-19 detection. Sci Total Environ.

[27] Yuan RYK, Li YQ, Han S, Chen XX, Chen JQ, He J, Gao HW, Yang Y, Yang SL, Yang Y (2022). Fe-curcumin nanozyme-mediated reactive oxygen species scavenging and anti-inflammation for acute lung injury. ACS Cent Sci.

[28] Wu F, Zhao S, Yu B, Chen Y-M, Wang W, Song Z-G, Hu Y, Tao Z-W, Tian J-H, Pei Y-Y (2020). A new coronavirus associated with human respiratory disease in China. Nature.

[29] Smyrlaki I, Ekman M, Lentini A, de Sousa NR, Papanicolaou N, Vondracek M, Aarum J, Safari H, Muradrasoli S, Rothfuchs AG, Albert J, Hogberg B, Reinius B (2020). Massive and rapid COVID-19 testing is feasible by extraction-free SARS-CoV-2 RT-PCR. Nat Commun.

[30] Huggett J, Dheda K, Bustin S, Zumla A (2005). Real-time RT-PCR normalisation; strategies and considerations. Genes Immun.

[31] Liu LH, Widen F, Baule C, Belak S (2007). A one-step, gel-based RT-PCR assay with comparable performance to real-time RT-PCR for detection of classical swine fever virus. J Virol Methods.

[32] Braunstein GD, Schwartz L, Hymel P, Fielding J (2021). False positive results with SARS-CoV-2 RT-PCR tests and how to evaluate a RT-PCR-positive test for the possibility of a false positive result. J Occup Environ Med.

[33] Jain PK, Huang X, El-Sayed IH, El-Sayed MA (2007). Review of some interesting surface plasmon resonance-enhanced properties of noble metal nanoparticles and their applications to biosystems. Plasmonics.

[34] Zada A, Muhammad P, Ahmad W, Hussain Z, Ali S, Khan M, Khan Q, Maqbool M (2020). Surface plasmonic-assisted photocatalysis and optoelectronic devices with noble metal nanocrystals: design, synthesis, and applications. Adv Func Mater.

[35] Chen Y, Ming H (2012). Review of surface plasmon resonance and localized surface plasmon resonance sensor. Photonic Sens.

[36] Solanki PR, Kaushik A, Agrawal VV, Malhotra BD (2011). Nanostructured metal oxide-based biosensors. NPG Asia Mater.

[37] Chen YP, Xianyu YL, Jiang XY (2017). Surface modification of gold nanoparticles with small molecules for biochemical analysis. Acc Chem Res.

[38] Bunz UHF, Rotello VM (2010). Gold nanoparticle-fluorophore complexes: sensitive and discerning “noses” for biosystems sensing. Angew Chem-Int Ed.

[39] Liu L, Jiang H, Wang XM (2021). Functionalized gold nanomaterials as biomimetic nanozymes and biosensing actuators. Trac-Trends Anal Chem.

[40] Masson JF (2017). Surface plasmon resonance clinical biosensors for medical diagnostics. Acs Sens.

[41] Sperling RA, Rivera Gil P, Zhang F, Zanella M, Parak WJ (2008). Biological applications of gold nanoparticles. Chem Soc Rev.

[42] Zhang X, Servos MR, Liu JW (2012). Instantaneous and quantitative functionalization of gold nanoparticles with thiolated DNA using a pH-assisted and surfactant-free route. J Am Chem Soc.

[43] De Fazio AF, Misatziou D, Baker YR, Muskens OL, Brown T, Kanaras AG (2021). Chemically modified nucleic acids and DNA intercalators as tools for nanoparticle assembly. Chem Soc Rev.

[44] Liu BW, Liu JW (2019). Interface-driven hybrid materials based on DNA-functionalized gold nanoparticles. Matter.

[45] Atapour A, Khajehzadeh H, Shafie M, Abbasi M, Mosleh-Shirazi S, Kasaee SR, Amani AM (2022). Gold nanoparticle-based aptasensors: a promising perspective for early-stage detection of cancer biomarkers. Mater Today Commun.

[46] Moitra P, Alafeef M, Dighe K, Frieman MB, Pan D (2020). Selective naked-eye detection of SARS-CoV-2 mediated by N gene targeted antisense oligonucleotide capped plasmonic nanoparticles. ACS Nano.

[47] Qiu G, Gai Z, Tao Y, Schmitt J, Kullak-Ublick GA, Wang J (2020). Dual-functional plasmonic photothermal biosensors for highly accurate severe acute respiratory syndrome coronavirus 2 detection. ACS Nano.

[48] Wang J, Drelich AJ, Hopkins CM, Mecozzi S, Li L, Kwon G, Hong S (2022). Gold nanoparticles in virus detection: recent advances and potential considerations for SARS-CoV-2 testing development. Wiley Interdiscip Rev Nanomed Nanobiotechnol.

[49] Tsang M-K, Ye W, Wang G, Li J, Yang M, Hao J (2016). Ultrasensitive detection of Ebola virus oligonucleotide based on upconversion nanoprobe/nanoporous membrane system. ACS Nano.

[50] Barrangou R, Doudna JA (2016). Applications of CRISPR technologies in research and beyond. Nat Biotechnol.

[51] Pickar-Oliver A, Gersbach CA (2019). The next generation of CRISPR—cas technologies and applications. Nat Rev Mol Cell Biol.

[52] Shivram H, Cress BF, Knott GJ, Doudna JA (2021). Controlling and enhancing CRISPR systems. Nat Chem Biol.

[53] Zhang WS, Pan J, Li F, Zhu M, Xu M, Zhu H, Yu Y, Su G (2021). Reverse transcription recombinase polymerase amplification coupled with CRISPR-Cas12a for facile and highly sensitive colorimetric SARS-CoV-2 detection. Anal Chem.

[54] ChrisáLe X (2021). CRISPR/Cas12a-mediated gold nanoparticle aggregation for colorimetric detection of SARS-CoV-2. Chem Commun.

[55] Patchsung M, Jantarug K, Pattama A, Aphicho K, Suraritdechachai S, Meesawat P, Sappakhaw K, Leelahakorn N, Ruenkam T, Wongsatit T (2020). Clinical validation of a Cas13-based assay for the detection of SARS-CoV-2 RNA. Nat Biomed Eng.

[56] Kudr J, Michalek P, Ilieva L, Adam V, Zitka O (2021). COVID-19: a challenge for electrochemical biosensors. Trac-Trends Anal Chem.

[57] Zhao Z, Huang CF, Huang ZY, Lin FJ, He QL, Tao D, Jaffrezic-Renault N, Guo ZZ (2021). Advancements in electrochemical biosensing for respiratory virus detection: a review. Trac-Trends Anal Chem.

[58] Rahman MM (2022). Progress in electrochemical biosensing of SARS-CoV-2 virus for COVID-19 management. Chemosensors.

[59] Vasquez V, Orozco J (2022). Detection of COVID-19-related biomarkers by electrochemical biosensors and potential for diagnosis, prognosis, and prediction of the course of the disease in the context of personalized medicine. Anal Bioanal Chem.

[60] Mahshid SS, Flynn SE, Mahshid S (2021). The potential application of electrochemical biosensors in the COVID-19 pandemic: a perspective on the rapid diagnostics of SARS-CoV-2. Biosens Bioelectron.

[61] Ji T, Liu Z, Wang G, Guo X, Lai C, Chen H, Huang S, Xia S, Chen B, Jia H (2020). Detection of COVID-19: a review of the current literature and future perspectives. Biosens Bioelectron.

[62] Maduraiveeran G, Sasidharan M, Ganesan V (2018). Electrochemical sensor and biosensor platforms based on advanced nanomaterials for biological and biomedical applications. Biosens Bioelectron.

[63] Privett BJ, Shin JH, Schoenfisch MH (2010). Electrochemical sensors. Anal Chem.

[64] Cho I-H, Kim DH, Park S (2020). Electrochemical biosensors: perspective on functional nanomaterials for on-site analysis. Biomater Res.

[65] Tripathy S, Singh SG (2020). Label-free electrochemical detection of DNA hybridization: a method for COVID-19 diagnosis. Trans Indian Natl Acad Eng.

[66] Peng Y, Pan Y, Sun Z, Li J, Yi Y, Yang J, Li G (2021). An electrochemical biosensor for sensitive analysis of the SARS-CoV-2 RNA. Biosens Bioelectron.

[67] Haun JB, Yoon TJ, Lee H, Weissleder R (2010). Magnetic nanoparticle biosensors. Wiley Interdiscip Rev-Nanomed Nanobiotechnol.

[68] Rocha-Santos TAP (2014). Sensors and biosensors based on magnetic nanoparticles. Trac-Trends Anal Chem.

[69] Jat SK, Gandhi HA, Bhattacharya J, Sharma MK (2021). Magnetic nanoparticles: an emerging nano-based tool to fight against viral infections. Mater Adv.

[70] Eivazzadeh-Keihan R, Bahreinizad H, Amiri Z, Aliabadi HAM, Salimi-Bani M, Nakisa A, Davoodi F, Tahmasebi B, Ahmadpour F, Radinekiyan F, Maleki A, Hamblin MR, Mahdavi M, Madanchi H (2021). Functionalized magnetic nanoparticles for the separation and purification of proteins and peptides. Trac-Trends Anal Chem.

[71] Zhao Z, Cui H, Song W, Ru X, Zhou W, Yu X (2020). A simple magnetic nanoparticles-based viral RNA extraction method for efficient detection of SARS-CoV-2. bioRxiv.

[72] Jang AS, Praveen Kumar PP, Lim D-K (2022). Attomolar sensitive magnetic microparticles and a surface-enhanced raman scattering-based assay for detecting SARS-CoV-2 nucleic acid targets. ACS Appl Mater Interfaces.

[73] Kim J, Mayorga-Martinez CC, Vyskočil J, Ruzek D, Pumera M (2022). Plasmonic-magnetic nanorobots for SARS-CoV-2 RNA detection through electronic readout. Appl Mater Today.

[74] Zayani R, Rezig D, Fares W, Marrakchi M, Essafi M, Raouafi N (2021). Multiplexed magnetofluorescent bioplatform for the sensitive detection of SARS-CoV-2 viral rna without nucleic acid amplification. Anal Chem.

[75] Juang DS, Juang TD, Dudley DM, Newman CM, Accola MA, Rehrauer WM, Friedrich TC, O’Connor DH, Beebe DJ (2021). Oil immersed lossless total analysis system for integrated RNA extraction and detection of SARS-CoV-2. Nat Commun.

[76] He Y, Lopez A, Zhang Z, Chen D, Yang R, Liu J (2019). Nucleotide and DNA coordinated lanthanides: from fundamentals to applications. Coord Chem Rev.

[77] Li Z, Chen X, Huang Z, Zhou J, Liu R, Lv Y (2021). Multiplex nucleic acid assay of SARS-CoV-2 via a lanthanide nanoparticle-tagging strategy. Anal Chem.

[78] Woo PCY, Lau SKP, Wong BHL, Tsoi HW, Fung AMY, Kao RYT, Chan KH, Peiris JSM, Yuen KY (2005). Differential sensitivities of severe acute respiratory syndrome (SARS) coronavirus spike polypeptide enzyme-linked immunosorbent assay (ELISA) and SARS coronavirus nucleocapsid protein ELISA for serodiagnosis of SARS coronavirus pneumonia. J Clin Microbiol.

[79] Peeling RW, Olliaro PL, Boeras DI, Fongwen N (2021). Scaling up COVID-19 rapid antigen tests: promises and challenges. Lancet Infect Dis.

[80] Dykman L, Khlebtsov N (2012). Gold nanoparticles in biomedical applications: recent advances and perspectives. Chem Soc Rev.

[81] Della Ventura B, Cennamo M, Minopoli A, Campanile R, Censi SB, Terracciano D, Portella G, Velotta R (2020). Colorimetric test for fast detection of SARS-CoV-2 in nasal and throat swabs. ACS Sens.

[82] Aithal S, Mishriki S, Gupta R, Sahu RP, Botos G, Tanvir S, Hanson RW, Puri IK (2022). SARS-CoV-2 detection with aptamer-functionalized gold nanoparticles. Talanta.

[83] Roberts A, Mahari S, Shahdeo D, Gandhi S (2021). Label-free detection of SARS-CoV-2 Spike S1 antigen triggered by electroactive gold nanoparticles on antibody coated fluorine-doped tin oxide (FTO) electrode. Anal Chim Acta.

[84] Zhou WZ, Huang PJJ, Ding JS, Liu J (2014). Aptamer-based biosensors for biomedical diagnostics. Analyst.

[85] Yang Y, Wu H, Liu B, Liu Z (2021). Tumor microenvironment-responsive dynamic inorganic nanoassemblies for cancer imaging and treatment. Adv Drug Deliv Rev.

[86] Pingarron JM, Yanez-Sedeno P, Gonzalez-Cortes A (2008). Gold nanoparticle-based electrochemical biosensors. Electrochim Acta.

[87] Anker JN, Hall WP, Lyandres O, Shah NC, Zhao J, Van Duyne RP (2008). Biosensing with plasmonic nanosensors. Nat Mater.

[88] Yang YJ, Murray J, Haverstick J, Tripp RA, Zhao YP (2022). Silver nanotriangle array based LSPR sensor for rapid coronavirus detection. Sens Actuators B-Chem.

[89] Bhalla N, Payam AF, Morelli A, Sharma PK, Johnson R, Thomson A, Jolly P, Canfarotta F (2022). Nanoplasmonic biosensor for rapid detection of multiple viral variants in human serum. Sens Actuators B Chem.

[90] Schlucker S (2014). Surface-enhanced raman spectroscopy: concepts and chemical applications. Angew Chem-Int Ed.

[91] Zhang Z, Li D, Wang XT, Wang YP, Lin JY, Jiang S, Wu Z, He YY, Gao X, Zhu Z, Xiao YL, Qu ZY, Li Y (2022). Rapid detection of viruses: based on silver nanoparticles modified with bromine ions and acetonitrile. Chem Eng J.

[92] Wu K, Saha R, Su DQ, Krishna VD, Liu JM, Cheeran MCJ, Wang JP (2020). Magnetic-nanosensor-based virus and pathogen detection strategies before and during COVID-19. ACS Appl Nano Mater.

[93] Zhong J, Rosch EL, Viereck T, Schilling M, Ludwig F (2021). Toward rapid and sensitive detection of SARS-CoV-2 with functionalized magnetic nanoparticles. ACS Sens.

[94] Wu K, Chugh VK, Krishna VD, di Girolamo A, Wang YA, Saha R, Liang S, Cheeran MCJ, Wang JP (2021). One-step, wash-free, nanoparticle clustering-based magnetic particle spectroscopy bioassay method for detection of SARS-CoV-2 spike and nucleocapsid proteins in the liquid phase. ACS Appl Mater Interfaces.

[95] Aminul Islam M, Ziaul Ahsan M (2020). Plausible approach for rapid detection of SARS-CoV-2 virus by magnetic nanoparticle based biosensors. Am J Nanosci.

[96] Durmus C, Harmanci D, Moulahoum H, Tok K, Ghorbanizamani F, Sanli S, Zihnioglu F, Evran S, Cicek C, Sertoz R, Arda B, Goksel T, Turhan K, Timur S, Hanoglu SB (2022). Indiscriminate SARS-CoV-2 multivariant detection using magnetic nanoparticle-based electrochemical immunosensing. Talanta.

[97] Azkur AK, Akdis M, Azkur D, Sokolowska M, van de Veen W, Bruggen MC, O'Mahony L, Gao YD, Nadeau K, Akdis CA (2020). Immune response to SARS-CoV-2 and mechanisms of immunopathological changes in COVID-19. Allergy.

[98] Zhang GX, Nie SK, Zhang ZH, Zhang ZT (2020). Longitudinal change of severe acute respiratory syndrome coronavirus 2 antibodies in patients with coronavirus disease 2019. J Infect Dis.

[99] Van Elslande J, Decru B, Jonckheere S, Van Wijngaerden E, Houben E, Vandecandelaere P, Indevuyst C, Depypere M, Desmet S, Andre E, Van Ranst M, Lagrou K, Vermeersch P (2020). Antibody response against SARS-CoV-2 spike protein and nucleoprotein evaluated by four automated immunoassays and three ELISAs. Clin Microbiol Infect.

[100] Lew TTS, Aung KMM, Ow SY, Amrun SN, Sutarlie L, Ng LFP, Su XD (2021). Epitope-functionalized gold nanoparticles for rapid and selective detection of SARS-CoV-2 IgG antibodies. ACS Nano.

[101] Pietschmann J, Voepel N, Vo L, Rasche S, Schubert M, Kleines M, Krause HJ, Shaw TM, Spiegel H, Schroeper F (2021). Development of fast and portable frequency magnetic mixing-based serological SARS-CoV-2-specific antibody detection assay. Front Macrobiol.

[102] Yadav S, Masud MK, Islam MN, Gopalan V, Lam KY, Tanaka S, Nguyen NT, Hossain M, Li C, Yamauchi YJN (2017). Gold-loaded nanoporous iron oxide nanocubes: a novel dispersible capture agent for tumor-associated autoantibody analysis in serum. Nanoscale.

[103] Gowri A, Ashwin Kumar N, Suresh Anand BS (2021). Recent advances in nanomaterials based biosensors for point of care (PoC) diagnosis of Covid-19—a minireview. TrAC Trends Anal Chem.

[104] Coronavirus disease (COVID-19) Pandemic—Emergency Use Listing Procedure (EUL) open for IVDs. https://extranet.who.int/pqweb/vitro-diagnostics/coronavirus-disease-covid-19-pandemic-%E2%80%94-emergency-use-listing-procedure-eul-open. Accessed 31 May 2022.

[105] Song Q, Sun X, Dai Z, Gao Y, Gong X, Zhou B, Wu J, Wen W (2021). Point-of-care testing detection methods for COVID-19. Lab Chip.

[106] Valera E, Jankelow A, Lim J, Kindratenko V, Ganguli A, White K, Kumar J, Bashir R (2021). COVID-19 point-of-care diagnostics: present and future. ACS Nano.

[107] Zhang Y, Malekjahani A, Udugama BN, Kadhiresan P, Chen H, Osborne M, Franz M, Kucera M, Plenderleith S, Yip L, Bader GD, Tran V, Gubbay JB, McGeer A, Mubareka S, Chan WCW (2021). Surveilling and tracking COVID-19 patients using a portable quantum dot smartphone device. Nano Lett.

[108] Beduk T, Beduk D, de Oliveira Filho JI, Zihnioglu F, Cicek C, Sertoz R, Arda B, Goksel T, Turhan K, Salama KN, Timur S (2021). Rapid point-of-care COVID-19 diagnosis with a gold-nanoarchitecture-assisted laser-scribed graphene biosensor. Anal Chem.

[109] Cheong J, Yu H, Lee CY, Lee J-U, Choi H-J, Lee J-H, Lee H, Cheon J (2020). Fast detection of SARS-CoV-2 RNA via the integration of plasmonic thermocycling and fluorescence detection in a portable device. Nat Biomed Eng.

[110] Bokelmann L, Nickel O, Maricic T, Pääbo S, Meyer M, Borte S, Riesenberg S (2021). Point-of-care bulk testing for SARS-CoV-2 by combining hybridization capture with improved colorimetric LAMP. Nat Commun.

[111] Li J, Lillehoj PB (2021). Microfluidic magneto immunosensor for rapid, high sensitivity measurements of SARS-CoV-2 nucleocapsid protein in serum. ACS Sens.

[112] COVID-19 Testing. What you need to know. https://www.cdc.gov/coronavirus/2019-ncov/symptoms-testing/testing.html. Accessed 31 May 2022.

[113] Wang C, Yang X, Gu B, Liu H, Zhou Z, Shi L, Cheng X, Wang S (2020). Sensitive and simultaneous detection of SARS-CoV-2-specific IgM/IgG using lateral flow immunoassay based on dual-mode quantum dot nanobeads. Anal Chem.

[114] Chen R, Ren C, Liu M, Ge X, Qu M, Zhou X, Liang M, Liu Y, Li F (2021). Early detection of SARS-CoV-2 seroconversion in humans with aggregation-induced near-infrared emission nanoparticle-labeled lateral flow immunoassay. ACS Nano.

[115] Huang C, Wen T, Shi F-J, Zeng X-Y, Jiao Y-J (2020). Rapid detection of IgM antibodies against the SARS-CoV-2 virus via colloidal gold nanoparticle-based lateral-flow assay. ACS Omega.

[116] Li Z, Yi Y, Luo X, Xiong N, Liu Y, Li S, Sun R, Wang Y, Hu B, Chen W, Zhang Y, Wang J, Huang B, Lin Y, Yang J, Cai W, Wang X, Cheng J, Chen Z, Sun K, Pan W, Zhan Z, Chen L, Ye F (2020). Development and clinical application of a rapid IgM-IgG combined antibody test for SARS-CoV-2 infection diagnosis. J Med Virol.

[117] Srivastav S, Dankov A, Adanalic M, Grzeschik R, Tran V, Pagel-Wieder S, Gessler F, Spreitzer I, Scholz T, Schnierle B, Anastasiou OE, Dittmer U, Schlücker S (2021). Rapid and sensitive SERS-based lateral flow test for SARS-CoV2-specific IgM/IgG antibodies. Anal Chem.

[118] Hsiao WWW, Sharma N, Le TN, Cheng YY, Lee CC, Vo DT, Hui YY, Chang HC, Chiang WH (2022). Fluorescent nanodiamond-based spin-enhanced lateral flow immunoassay for detection of SARS-CoV-2 nucleocapsid protein and spike protein from different variants. Anal Chim Acta.

[119] Chen ZH, Zhang ZG, Zhai XM, Li YY, Lin L, Zhao H, Bian L, Li P, Yu L, Wu YS (2020). Rapid and sensitive detection of anti-SARS-CoV-2 IgG, using lanthanide-doped nanoparticles-based lateral flow immunoassay. Anal Chem.

[120] Telenti A, Arvin A, Corey L, Corti D, Diamond MS, Garcia-Sastre A, Garry RF, Holmes EC, Pang PS, Virgin HW (2021). After the pandemic: perspectives on the future trajectory of COVID-19. Nature.

[121] Diamond MS, Lambris JD, Ting JP, Tsang JS (2022). Considering innate immune responses in SARS-CoV-2 infection and COVID-19. Nat Rev Immunol.

[122] Dorlass EG, Lourenco KL, Magalhaes RDM, Sato H, Fiorini A, Peixoto R, Coelho HP, Telezynski BL, Scagion GP, Ometto T, Thomazelli LM, Oliveira DBL, Fernandes AP, Durigon EL, Fonseca FG, Teixeira SMR (2021). Survey of SARS-CoV-2 genetic diversity in two major Brazilian cities using a fast and affordable Sanger sequencing strategy. Genomics.

[123] Clark AE, Wang ZH, Ostman E, Zheng H, Yao HY, Cantarel B, Kanchwala M, Xing C, Chen L, Irwin P, Xu Y, Oliver D, Lee FM, Gagan JR, Filkins L, Muthukumar A, Park JY, Sarode R, SoRelle JA (2022). Multiplex fragment analysis for flexible detection of all SARS-CoV-2 variants of concern. Clin Chem.

[124] Van Poelvoorde LAE, Delcourt T, Coucke W, Herman P, De Keersmaecker SCJ, Saelens X, Roosens NHC, Vanneste K (2021). Strategy and performance evaluation of low-frequency variant calling for SARS-CoV-2 using targeted deep illumina sequencing. Front Microbiol.

[125] Dachert C, Muenchhoff M, Graf A, Autenrieth H, Bender S, Mairhofer H, Wratil PR, Thieme S, Krebs S, Grzimek-Koschewa N, Blum H, Keppler OT (2022). Rapid and sensitive identification of omicron by variant-specific PCR and nanopore sequencing: paradigm for diagnostics of emerging SARS-CoV-2 variants. Med Microbiol Immunol.

[126] Ko KKK, Rahman NBA, Tan SYL, Chan KXL, Goh SS, Sim JHC, Lim KL, Tan WL, Chan KS, Oon LLE, Nagarajan N, Suphavilai C (2022). SARS-CoV-2 N gene G29195T point mutation may affect diagnostic reverse transcription-PCR detection. Microbiol Spectr.

[127] Corman VM, Landt O, Kaiser M, Molenkamp R, Meijer A, Chu DKW, Bleicker T, Brunink S, Schneider J, Schmidt ML, Mulders D, Haagmans BL, van der Veer B, van den Brink S, Wijsman L, Goderski G, Romette JL, Ellis J, Zambon M, Peiris M, Goossens H, Reusken C, Koopmans MPG, Drosten C (2020). Detection of 2019 novel coronavirus (2019-nCoV) by real-time RT-PCR. Eurosurveillance.

[128] Rosato AE, Msiha E, Weng B, Mesisca M, Gnass R, Gnass S, Bol C, Tabuenca A, Rosato RR (2022). Rapid detection of the widely circulating B.1.617.2 (Delta) SARS-CoV-2 variant. Pathology.

[129] Dikdan RJ, Marras SAE, Field AP, Brownlee A, Cironi A, Hill DA, Tyagi S (2022). Multiplex PCR assays for identifying all major severe acute respiratory syndrome coronavirus 2 variants. J Mol Diagn.

[130] Durand M, Thibault P, Levesque S, Brault A, Carignan A, Valiquette L, Martin P, Labbe S (2022). Detection of severe acute respiratory syndrome coronavirus 2 (SARS-CoV-2) and its first variants in fourplex real-time quantitative reverse transcription-PCR assays. Microbial Cell.

[131] Luo Z, Ye CH, Xiao H, Yin JL, Liang YC, Ruan ZH, Luo DJ, Gao DL, Tan QP, Li YK, Zhang QW, Liu WY, Wu JG (2022). Optimization of loop-mediated isothermal amplification (LAMP) assay for robust visualization in SARS-CoV-2 and emerging variants diagnosis. Chem Eng Sci.

[132] Xiao HY, Hu JY, Huang C, Feng W, Liu YM, Kumblathan T, Tao J, Xu JY, Le XC, Zhang HQ (2023). CRISPR techniques and potential for the detection and discrimination of SARS-CoV-2 variants of concern. Trac-Trends Anal Chem.

[133] Renzoni A, Perez F, Nsoga MTN, Yerly S, Boehm E, Gayet-Ageron A, Kaiser L, Schibler M (2021). Analytical evaluation of visby medical RT-PCR portable device for rapid detection of SARS-CoV-2. Diagnostics.

[134] Chen Z, Li J, Li T, Fan T, Meng C, Li C, Kang J, Chai L, Hao Y, Tang Y (2022). A CRISPR/Cas12a-empowered surface plasmon resonance platform for rapid and specific diagnosis of the Omicron variant of SARS-CoV-2. Natl Sci Rev.

[135] Marques MC, Ruiz R, Montagud-Martinez R, Marquez-Costa R, Albert S, Domingo-Calap P, Rodrigo G (2021). CRISPR-Cas12a-based detection of SARS-CoV-2 harboring the E484K mutation. ACS Synth Biol.

[136] Ali Z, Sanchez E, Tehseen M, Mahas A, Marsic T, Aman R, Rao GS, Alhamlan FS, Alsanea MS, Al-Qahtani AA (2022). Bio-SCAN: a CRISPR/dCas9-based lateral flow assay for rapid, specific, and sensitive detection of SARS-CoV-2. ACS Synth Biol.

[137] Arizti-Sanz J, Bradley A, Zhang YB, Boehm CK, Freije CA, Grunberg ME, Kosoko-Thoroddsen TSF, Welch NL, Pillai PP, Mantena S (2022). Simplified Cas13-based assays for the fast identification of SARS-CoV-2 and its variants. Nat Biomed Eng.

[138] de Puig H, Lee RA, Najjar D, Tan X, Soekensen LR, Angenent-Mari NM, Donghia NM, Weckman NE, Ory A, Ng CF (2021). Minimally instrumented SHERLOCK (miSHERLOCK) for CRISPR-based point-of-care diagnosis of SARS-CoV-2 and emerging variants. Sci Adv.

[139] Khalid MF, Selvam K, Jeffry AJN, Salmi MF, Najib MA, Norhayati MN, Aziah I (2022). Performance of rapid antigen tests for COVID-19 diagnosis: a systematic review and meta-analysis. Diagnostics.

[140] Jiang WJ, Ji WQ, Zhang Y, Xie YQ, Chen SY, Jin YF, Duan GC (2022). An update on detection technologies for SARS-CoV-2 variants of concern. Viruses-Basel.

[141] Medoro A, Davinelli S, Voccola S, Cardinale G, Passarella D, Marziliano N, Intrieri M (2022). Assessment of the diagnostic performance of a novel SARS-CoV-2 antigen sealing tube test strip (colloidal gold) as point-of-care surveillance test. Diagnostics.

[142] Ollier Q, Pillet S, Mory O, Gagnaire J, Thuiller C, Annino N, Gagneux-Brunon A, Botelho-Nevers E, Bourlet T, Pozzetto B, Cantais A (2022). Prospective evaluation of the point-of-care use of a rapid antigenic SARS-CoV-2 immunochromatographic test in a paediatric emergency department. Clin Microbiol Infect.

[143] Takeuchi Y, Akashi Y, Kiyasu Y, Terada N, Kurihara Y, Kato D, Miyazawa T, Muramatsu S, Shinohara Y, Ueda A, Notake S, Nakamura K, Suzuki H (2022). A prospective evaluation of diagnostic performance of a combo rapid antigen test QuickNavi-Flu+COVID19 Ag. J Infect Chemother.

[144] Osterman A, Badell I, Basara E, Stern M, Kriesel F, Eletreby M, Oztan GN, Huber M, Autenrieth H, Knabe R, Spath PM, Muenchhoff M, Graf A, Krebs S, Blum H, Durner J, Czibere L, Dachert C, Kaderali L, Baldauf HM, Keppler OT (2022). Impaired detection of omicron by SARS-CoV-2 rapid antigen tests. Med Microbiol Immunol.

[145] Dinnes J, Deeks JJ, Berhane S, Taylor M, Adriano A, Davenport C, Dittrich S, Emperador D, Takwoingi Y, Cunningham J, Beese S, Domen J, Dretzke J, di Ruffano LF, Harris IM, Price MJ, Taylor-Phillips S, Hooft L, Leeflang MMG, McInnes MD, Spijker R, Van den Bruel A, Cochrane C-DTA (2021). Rapid, point-of-care antigen and molecular-based tests for diagnosis of SARS-CoV-2 infection. Cochrane Database of Syst Rev.

[146] Xu C, Lei C, Hosseinpour S, Ivanovski S, Walsh LJ, Khademhosseini A (2022). Nanotechnology for the management of COVID-19 during the pandemic and in the post-pandemic era. Natl Sci Rev.

